# Integrated Metabolomic and Proteomic Analyses Reveal Differential Regulation of the Citrate Cycle and Pentose Phosphate Pathway by *Cannabis sativa* Extract and the Synthetic Cannabinoid HU210 in GT1-7 Neuronal Cells: Sequential Extraction of Metabolites and Proteins from a Single Cell Pellet

**DOI:** 10.3390/metabo16090645

**Published:** 2026-09-03

**Authors:** Yujin Natori, Dai Mizuno, Masaru Doshi, Masahiro Kawahara, Akira Ishii

**Affiliations:** 1 Department of Legal Medicine and Bioethics, Nagoya University Graduate School of Medicine, 65 Tsurumai-cho, Showa-ku, Nagoya-shi 466-8550, Japan; akishii@med.nagoya-u.ac.jp; 2 Department of Forensic Medicine, Faculty of Medicine, Yamagata University, 2-2-2 Iida-Nishi, Yamagata-shi 990-9585, Japan; d_mizuno@med.id.yamagata-u.ac.jp; 3 Department of Human Physiology and Pathology, Faculty of Pharmaceutical Sciences, Teikyo University, 2-11-1 Kaga, Itabashi-ku 173-8605, Japan; doshi.masaru.ww@teikyo-u.ac.jp; 4 Research Institute of Pharmaceutical Sciences, Faculty of Pharmacy, Musashino University, 1-1-20 Shin-machi, Nishitokyo-shi 202-8585, Japan; makawa@musashino-u.ac.jp

**Keywords:** *Cannabis sativa*, synthetic cannabinoids, HU210, GT1-7, neuroblastoma, metabolomics, proteomics, integrated omics

## Abstract

**Highlights:**

**What are the main findings?**

- HU210 altered both the metabolome and proteome, whereas *Cannabis* extract altered the metabolome with minimal proteome change in GT1-7 cells.
- Five pathways—citrate cycle, pentose phosphate pathway, pyruvate, butanoate, and glycerolipid metabolism—were differentially regulated by HU210 and *Cannabis* extract.

**What are the implications of the findings?**

- Distinct metabolome–proteome response patterns indicate that HU210 and *Cannabis* extract produce different early metabolic and proteomic responses.
- Metabolite changes occurring without corresponding proteome changes may represent an early signal of cellular stress that precedes detectable changes in protein abundance.

**Abstract:**

Background: *Cannabis* contains Δ^9^-tetrahydrocannabinol (THC) together with numerous other constituents, whereas HU210 (HU) is a synthetic full agonist at cannabinoid receptors. We applied integrated metabolomics and proteomics to compare *Cannabis* extract (CS) and HU in GT1-7 neuronal cells. Methods: Cytotoxicity was evaluated using a lactate dehydrogenase assay. Metabolites and proteins were extracted sequentially from single-cell pellets and analyzed using gas chromatography–tandem mass spectrometry and nano-liquid chromatography–quadrupole–Orbitrap mass spectrometry, respectively. Cannabinoids in the exposure medium were quantified using liquid chromatography–tandem mass spectrometry. Results: The lowest CS and HU concentrations that were cytotoxic at 24 h and non-cytotoxic at 3 h were 2000 µg/mL and 20 µM, respectively; CS delivered 166 nM THC, 800 nM tetrahydrocannabinolic acid, and 11.3 nM cannabidiol. After 3 h, HU altered both the metabolome and the proteome, whereas CS altered the metabolome with minimal proteome change (8 of 2720 proteins). Six pathways differed, including the citrate cycle and pentose phosphate pathway. Relative to both the control and CS, HU decreased citrate; increased 2-ketoglutarate, fumarate, malate, gluconate, ribose-5-phosphate and ribose; and increased phosphoenolpyruvate carboxykinase 2, isocitrate dehydrogenase 3 subunit α, 2-oxoglutarate dehydrogenase complex component E1, succinate dehydrogenase flavoprotein subunit, malate dehydrogenase 1, 6-phosphogluconolactonase, 6-phosphogluconate dehydrogenase and phosphoglucomutase-1. CS showed the opposite metabolite changes without corresponding enzyme changes. Conclusions: At a pre-lethal time point, CS and HU produced metabolic changes in opposite directions—broad suppression versus coordinated mobilisation—despite comparable cytotoxicity at 24 h.

## 1. Introduction

The endocannabinoid system (ECS) is a lipid-based neuromodulatory network that regulates a broad range of physiologic functions, including pain, appetite, mood, memory, immune surveillance, and neuronal plasticity throughout life, beginning at the earliest stages of brain development [1,2]. The ECS comprises cannabinoid (CB) receptors, their endogenous ligands (endocannabinoids), and the enzymes responsible for their biosynthesis and degradation [1]. The first molecular characterization of the ECS was conducted by Devane and colleagues, who in 1988 identified a specific CB binding site in rat brain membranes [3]. This led to the cloning of two pharmacologically distinct receptor subtypes: cannabinoid receptor type 1 (CB1R) and type 2 (CB2R), both of which are class A G protein-coupled receptors (GPCRs) [4]. CB1R is the most abundantly expressed GPCR in the mammalian central nervous system (CNS), concentrated in the basal ganglia, hippocampus, cerebral cortex, and cerebellum [5]. Localized predominantly to presynaptic terminals, CB1R activation suppresses neurotransmitter release via Gi/o protein-mediated inhibition of adenylyl cyclase, modulation of voltage-gated calcium channels, and activation of inwardly rectifying potassium channels [2,4]. CB2R was initially described as a peripheral receptor on immune cells regulating cytokine secretion; however, it has also been identified in the CNS under neuroinflammatory conditions and implicated in neuroprotection mechanisms [2,5]. The principal endocannabinoids, *N*-arachidonoylethanolamine (anandamide, AEA) and 2-arachidonoylglycerol (2-AG), are synthesized on demand, act as retrograde synaptic messengers, and are rapidly hydrolyzed [6,7]. Endocannabinoids also engage additional targets beyond CB1R and CB2R, including transient receptor potential vanilloid 1 (TRPV1), G protein-coupled receptor 55 (GPR55), and peroxisome proliferator-activated receptor gamma (PPARγ). The various receptors affected by endocannabinoids illustrate the breadth of the expanded ECS signaling network [4,8].

*Cannabis sativa* contains over 400 chemical entities, more than 60 of which are classified as phytocannabinoids [5]. Δ^9^-Tetrahydrocannabinol (THC), the principal psychoactive constituent, acts primarily as a low-efficacy partial agonist at the CB1R, resulting in euphoria; however, it can also cause memory impairment and, at higher doses, anxiety, by engaging CB1Rs in the cortex, hippocampus, amygdala, and striatum [5,8,9]. Cannabidiol (CBD), which can constitute up to 40% of the CB content in some chemotypes, is non-intoxicating and exerts neuroprotective, anxiolytic, and anti-inflammatory effects, partly by interacting with TRPV1, serotonin receptor 1A (5-HT1A), and GPR55 rather than directly binding to CB1R or CB2R [5,8]. Importantly, CBD can counteract the adverse effects of THC, such as anxiety and cognitive impairment, illustrating the pharmacologic interplay among *Cannabis* constituents [9].

The therapeutic potential of *Cannabis* extends beyond any single compound. Russo formalized the concept of the “phytocannabinoid-terpenoid entourage effect” and proposed that minor cannabinoids, terpenes, and flavonoids interact synergistically to produce effects that exceed those of any purified component [10]. For instance, β-caryophyllene, a terpene, is a confirmed dietary CB2R agonist, and experimental data indicate that full-spectrum extracts of *Cannabis* outperform equivalent doses of isolated THC [10]. The neurological consequences of *Cannabis sativa* extract (CS) exposure therefore arise from highly integrated polypharmacologic interactions that engage multiple receptor systems simultaneously, generating complex intracellular cascades that remain incompletely characterized, particularly at the level of the neuronal proteome and metabolome.

Synthetic CBs constitute a structurally heterogeneous class designed to mimic and often substantially exceed the receptor-binding properties of THC [9]. Among these compounds, HU210 (HU) [(–)-1,1-dimethylheptyl-11-hydroxy-Δ^8^-tetrahydrocannabinol], first synthesized in 1988, is one of the most potent CB receptor agonists available [11]. HU acts as a full agonist upon both CB1R (Ki = 0.061 nM) and CB2R (Ki = 0.52 nM), exhibiting 100- to 800-fold greater potency than THC in behavioral assays, with a markedly prolonged duration of action [11,12]. Crucially, as a full agonist, HU achieves maximal Go protein activation at CB1R, which the partial agonist THC cannot, a distinction with fundamental implications for downstream signaling [13]. HU engages several key intracellular signaling cascades through CB1R and CB2R: inhibition of adenylyl cyclase, activation of the MAPK/ERK and PI3K/AKT pathways, and modulation of ion channel conductance [12,14]. The PI3K/AKT pathway mediates HU-induced neuroprotection, as demonstrated in primary cortical neurons subjected to S-enantiomer α-amino-3-hydroxy-5-methyl-4-isoxazolepropionic acid-induced excitotoxicity [14]. By contrast, HU also impairs spatial memory and suppresses the firing rate of hippocampal neurons, which correlates with region-specific CB1R downregulation [15], and repeated administration of HU generates antidepressant-like effects via hippocampal CB1R-dependent mechanisms [16]. These paradoxical outcomes underscore the dose-, regimen-, and brain region-dependent nature of HU activity and qualitatively distinguish HU from THC as a tool for delineating maximal CB receptor-mediated neurochemical responses.

Available evidence indicates that reductionistic single-endpoint approaches are insufficient to comprehensively characterize the molecular impact of CB exposure on neurons. High-throughput omics technologies now enable the simultaneous quantification of thousands of biological molecules, providing a systems-level view of cellular pharmacology [17]. Metabolomics, the global profiling of small molecules, enables the capture of real-time snapshots of energy metabolism, neurotransmitter turnover, lipid mediator production, and oxidative stress [17,18]. Proteomics provides complementary information regarding protein abundance, post-translational modifications, and signaling pathway activation that cannot be inferred from transcriptomic data alone [19]. Integrating these two platforms—a cornerstone of multi-omics systems biology research—enables simultaneous investigation of processes at multiple biological levels, thereby revealing molecular networks and regulatory nodes that underlie a given phenotype of interest [17,19,20]. Specifically, joint proteome/metabolome analyses can identify rate-limiting enzymatic steps in perturbed pathways by correlating enzyme abundance with substrate and product levels, thus providing mechanistic depth that neither platform alone can achieve [18,19].

In the present study, we applied an integrated metabolomics and proteomics approach to investigate the effects of CS and HU on GT1-7 immortalized hypothalamic neuronal cells expressing both CB1R and CB2R [21,22]. Multi-omics profiles induced by the pharmacologically complex CS were compared with those induced by the selective full-agonist HU with the aim of disentangling responses by the pure synthetic CB HU from entourage-driven interactions in CS containing complex components. Furthermore, metabolites and proteins extracted from the same cell pellets were examined using integrated omics analyses, which enabled the identification of direct correlations between molecular profiles across metabolome and proteome layers within the same sample. The resulting identification of differentially regulated proteins and metabolites advances current understanding of the molecular mechanisms underlying the toxicity of synthetic CB and *Cannabis* products and could facilitate the development of novel ECS-targeted therapeutic strategies.

## 2. Materials and Methods

### 2.1. Preparation of CS and Qualification and Quantification of CBs Using Liquid Chromatography–Tandem Mass Spectrometry

*Cannabis sativa* from Mexico was kindly provided as a gift by the late Dr. Ikuo Yamamoto (Kyushu University of Medical Science, Miyazaki, Japan). The plant material was weighed (100 mg), frozen in liquid nitrogen, and then processed using a multi-bead shocker (Yasui Kikai Corp., Osaka, Japan). The samples were subsequently extracted using 1 mL of chloroform (FUJIFILM Wako Pure Chemicals Corporation., Osaka, Japan) for 1 h at 4 °C with rotation. After centrifugation at 10,000× *g* for 5 min at 4 °C, the supernatant was transferred to a new tube and evaporated under a gentle nitrogen flow in a draft chamber equipped with a HEPA filter. The dried residue was reconstituted in 100 µL of dimethyl sulfoxide (DMSO) cell culture reagent (Santa Cruz Biotechnology, Inc., Dallas, TX, USA), and insoluble particles were removed using centrifugation. The solution was prepared as a stock CS and stored at −80 °C until use. Concentrations of CS are expressed throughout as micrograms of dried plant material equivalent per milliliter of culture medium; the stock solution corresponded to 1 mg of dried plant material per microliter of DMSO. To qualify and quantify the CB content of CS samples, the residue was reconstituted with the primary mobile phase described below, and insoluble residues were removed using centrifugation and filtration through a Millex GV syringe-driven filter (PVDF, 0.22 µm, 4 mm) (Merck KGaA, Darmstadt, Germany). The specimens and authentic CBs were analyzed on an LCMS8050 liquid chromatography–tandem mass spectrometry system (Shimadzu Corporation., Kyoto, Japan) coupled with a Nexera X2 system (Shimadzu Corporation). The following authentic CBs were analyzed: cannabichromene (CBC), cannabidiol (CBD), tetrahydrocannabivarin (THCV), tetrahydrocannabinolic acid (THCA), cannabicycrol (CBL), and cannabicyclic acid (CBLA), which were purchased from Cerilliant Corporation. (Round Rock, TX, USA). THC was obtained from the late Dr. Ikuo Yamamoto as described above for *Cannabis sativa* plants. A Raptor ARC-18 column (particle size 2.7 µm, 150 mm × 2.1 mm, Restek Corporation, Bellefonte, PA, USA) equipped with a Raptor ARC-18 guard cartridge (particle size 2.7 µm, 5 mm × 2.1 mm, Restek Corporation) and an EXP Direct connect holder (Restek Corporation) was used for separation. The flow rate was 0.3 mL/min, and a binary gradient was employed for elution. The aqueous phase was 0.1% formate (FUJIFILM WAKO Pure Chemicals, Corporation) and 5 mM ammonium formate (Sigma-Aldrich Co. LLC, St. Louis, MO, USA) in ultra-pure water (UW) (mobile phase A), and the organic phase was 0.1% formate in acetonitrile (FUJIFILM WAKO Pure Chemicals Corporation) (mobile phase B). Elution was performed as follows: 75% B from 0 to 6 min, 75% to 85% B from 6.1 to 7.5 min, 100% B from 7.6 to 9.0, then 75% B from 9.1 to 14 min. The precursor ions, retention times, collision energies, and mass spectra for each CB in the CS and authentic samples are shown in Supplementary Figure S1. To quantify CBD, THC, and THCA, we employed the standard addition method to minimize matrix effects and improve quantification accuracy. For the analysis, CS extract was added at concentrations of 0.125 mg/mL, 1.5 mg/mL, and 25 mg/mL to the primary mobile phase to quantify THCA, THC, and CBD, respectively. The concentrations of the calibration curve were 0, 15.6, 31.2, 46.9, and 62.5 ng/mL. THC-*d*3 was used as the internal standard at 31.25 ng/mL. The correlation coefficients for each target analyte were >0.99. The results are shown in Supplementary Table S4.

### 2.2. Cell Culture and Incubation with CS and the Synthetic CB HU

GT1-7 mouse hypothalamus-derived neuroblastoma cells were kindly provided by Dr. Richard Weiner (University of California, San Francisco, San Francisco, CA, USA). The cells were cultured in Dulbecco’s modified Eagle’s medium/F-12HAM (FUJIFILM Wako Pure Chemicals Corporation) containing 10% fetal bovine serum (FBS) (Biowest, Nuaillé, France) and 1% penicillin and streptomycin (FUJIFILM WAKO Pure Chemicals Corporation) (culture medium). The FBS was heat-inactivated by incubation at 56 °C for 30 min. The cells were used for no more than 10 passages. Cells were maintained at 37 °C with 5% CO_2_. For experiments, cells were seeded in culture dishes with medium at 5 × 10^4^ cells/cm^2^ and incubated overnight. The next day, the cells were incubated with CS or the synthetic CB HU (HU210, Tokyo Chemical Industry Co., Ltd., Tokyo, Japan) at the concentrations indicated in the Results Section. Stock HU solution was prepared in DMSO and stored at −30 °C.

When the DMSO stock of CS was added to the culture medium, a visible precipitate formed. The medium was therefore centrifuged at 1000× *g* for 3 min at 25 °C and the supernatant used for exposure. The concentration of 2000 µg/mL accordingly refers to the nominal amount of plant material equivalent added. The same medium preparation procedure was used for the lactate dehydrogenase (LDH) assays and for the metabolomic and proteomic experiments.

Because CS is a multi-component botanical preparation and HU is a single synthetic compound, equimolar comparison between the two is not chemically meaningful. Exposure concentrations were therefore selected on an iso-effective basis: for each substance, we used the lowest concentration that produced significant LDH release at 24 h and that was not cytotoxic at 3 h. This design matches the two exposures on the toxicological endpoint of interest rather than on chemical concentration.

The cytotoxicity of CS and HU was evaluated using a Cytotoxicity LDH Assay kit-WST (Dojindo Laboratories, Kumamoto, Japan) according to the manufacturer’s protocol. Cells were seeded in a 96-well culture plate and incubated for 3 and 24 h in the presence of the indicated concentrations of CS and HU. The medium was then transferred to a new 96-well plate containing assay reagent in each well, and the absorbance of each well at 490 nm was measured using a MULTISKAN SkyHigh microplate reader (Thermo Fisher Scientific Inc., Waltham, MA, USA) (*n* = 4 per group). The data were analyzed statistically using R (ver. 4. 4. 1) and RStudio (2026. 01) and compared using one-way analysis of variance (ANOVA) and Tukey’s post hoc test (alpha = 0.05). Cytotoxicity was evaluated by measuring the absorbance at 490 nm.

### 2.3. Extraction Procedure and Metabolomic Analysis

Cells were incubated in a 6-well plate for 3 h with 2000 µg/mL CS or 20 µM HU in the culture medium. Cells were washed twice with ice-cold Dulbecco’s phosphate-buffered saline (DPBS; FUJIFILM Wako Pure Chemicals Corporation) and harvested on a Coolbox XT (WAKEN BTECH Co., Ltd., Kyoto, Japan) using a cell scraper in 700 µL/well of ice-cold methanol (FUJIFILM Wako Pure Chemicals Corporation) containing 0.5 µg/mL glutamic acid-^13^C_5_, ^15^N as an internal standard (IS; Toronto Research Chemicals Inc., Vaughan, ON, Canada), and immediately frozen in liquid nitrogen. The cell and methanol fractions were examined as metabolomic samples and stored at −80 °C until analysis (*n* = 18 per group).

The samples were subjected to 3 cycles of thawing and freezing in liquid nitrogen for 2 min, followed by 2 min of mixing at 1400 rpm and 37 °C using a TS-100C (SIA BIOSAN, Riga, Latvia) equipped with an SC-24NC block for 24 × 1.5 mL microtubes to extract the metabolome. The samples were then centrifuged at 15,000× *g* for 5 min at 4 °C. The resulting supernatants were transferred to new tubes, and the pellets were used to compensate for the metabolome based on protein concentration and to extract the proteome. A 200 µL aliquot of each supernatant was mixed with 100 µL of chloroform and 100 µL of UW (FUJIFILM Wako Pure Chemicals Corporation); alternatively, 10 µL of each supernatant was mixed with 190 µL of methanol and the same volume of chloroform and UW indicated above. The resulting mixtures were mixed at 1400 rpm and 4 °C for 10 min and then centrifuged at 15,000× *g* at 4 °C for 5 min. A total of 250 µL of the aqueous phase was transferred to a new tube, and 250 µL of UW was added; the mixture was incubated and centrifuged as described above. Next, 450 µL of supernatant was transferred to a new tube, and the organic solvent was evaporated using a CentriVap vacuum centrifuge (Labconco Corporation, Kansas City, MO, USA) at ambient temperature for 1 h. The samples were then frozen in liquid nitrogen for 5 min and freeze-dried overnight using an EYELA FDU-1200 freeze dryer (TOKYO RIKAKIKAI Co., Ltd., Tokyo, Japan).

The next day, the dried samples were reconstituted with 40 µL of 20 mg/mL methoxyamine hydrochloride (Sigma-Aldrich Co. LLC) in pyridine (FUJIFILM Wako Pure Chemicals Corporation) and sonicated for 20 min using a SUS USK-4R sonicator (As One Corp., Osaka, Japan). After sonication, the samples were mixed at 1400 rpm and 30 °C for 90 min. For trimethylsilyl derivatization, 20 µL of *N*-methyl-*N*-trimethylsilyl trifluoroacetamide (Sigma-Aldrich Co. LLC) was added to each sample, followed by mixing at 1400 rpm and 37 °C for 30 min. Finally, 1 µL of each sample was analyzed using gas chromatography–tandem mass spectrometry on a GCTQ8040 instrument (Shimadzu Corporation.) equipped with a DB-5 capillary column (30 m × 0.25 mm id, df = 1.00 μm, Agilent Technologies, Inc., Santa Clara, CA, USA). Samples were injected in splitless mode. The oven was initially set to 100 °C for 4 min, then heated linearly to 320 °C at 10 °C/min and held at 320 °C for 11 min. The carrier gas was high-purity helium gas, and the flow rate was 1.1 mL/min. Electron ionization was carried out at 70 eV. Selective reaction monitoring was used to detect and quantify the metabolites. Data acquisition was performed with GCMS solution software (version 4.60 SP1, Shimadzu Corporation.).

The area under the curve of the corresponding chromatogram (AUC) for each analyte was normalized against the AUC of the IS and the protein concentration of the sample pellet. Three quality control (QC) samples were prepared by mixing equal volumes of the samples measured in the same batch, and these samples were analyzed every 7 data acquisitions. Metabolites with a relative standard deviation < 25% across the 3 QC samples were used for subsequent analyses (75 metabolites; Supplementary Table S2).

MetaboAnalyst 6.0 software was used to generate principal component analysis (PCA) score plots, heatmaps, and evaluate the data using one-way analysis of variance (ANOVA) with Tukey’s post hoc test. Data were log_2_-transformed, and Pareto scaling was applied. Statistical significance was defined as a false discovery rate (FDR) of <0.05 in one-way ANOVA.

### 2.4. Preparation and Analysis of the Proteome

The cell pellet obtained during metabolome extraction was lysed in EasyPep Lysis buffer (Thermo Fisher Scientific Inc.) and centrifuged at 10,000× *g* and 4 °C for 5 min. The resulting supernatant was then transferred to a new tube, and the protein concentration was determined using a TAKARA BCA protein assay kit (Takara Bio Inc., Shiga, Japan). The samples were then adjusted to 60 µg/100 µL using the same buffer indicated above and prepared using an EasyPep mini MS sample prep kit (Thermo Fisher Scientific Inc.) according to the manufacturer’s protocol. Before proteome analysis, the samples were subjected to sodium dodecyl sulfate–polyacrylamide gel electrophoresis to ensure equal protein amounts across the samples.

Peptides were analyzed using a Q-Exactive mass spectrometer (Thermo Fisher Scientific Inc.) and an UltiMate3000 RSLCnano LC system (Dionex Co., Ltd., Amsterdam, The Netherlands) with a nano-high-performance liquid chromatography capillary column (150 mm × 75 μm i.d.; Nikkyo Technos Co., Ltd., Tokyo, Japan) via a nanoelectrospray ion source. The analyses were conducted by the Division for Medical Research Engineering of our university. A precursor ion scan was carried out over a mass-to-charge ratio (*m*/*z*) range of 400 to 600 prior to MS2 analysis. MS2 scans were obtained for the 20 most intense peaks of each MS1 scan.

Raw data were processed using Proteome Discoverer, version 2.4 (Thermo Fisher Scientific Inc.) in conjunction with the MASCOT search engine, version 3.1.0 (Matrix Science Inc., Boston, MA, USA), for protein identification. Peptides and proteins were identified against the mouse protein database in UNIPROT (release 2026_01), with a precursor mass tolerance of 10 ppm and fragment ion mass tolerance of 0.02 Da. Fixed modification was set to carbamidomethylation of cysteine, and variable modifications were set to oxidation of methionine and acetylation of the peptide N-terminus. Two missed cleavages by trypsin were allowed. Protein abundance was normalized to the total abundance in each sample. Appropriate proteomic datasets subjected to all analyses were selected according to the workflows shown in Supplementary Figure S2.

MetaboAnalyst 6.0 was used to generate PCA score plots and heatmaps, and data were analyzed using one-way ANOVA with Tukey’s post hoc test. Details regarding data transformation and scaling were the same as those described above for the metabolomic analysis (*n* = 6 per group).

### 2.5. Metabolome and Proteome Integrated Pathway Analysis

Integrated pathway analysis of the metabolome and proteome was performed using MetaboAnalyst 6.0. The input metabolome included 75 metabolites, and the proteome included 256 of 267 significant proteins determined using one-way ANOVA with Tukey’s post hoc test (the proteins used in the analysis were annotated via joint pathway analysis using MetaboAnalyst 6.0); joint pathway analysis was employed to identify significantly enriched pathways. Pathway identification was carried out using *Mus musculus* as the animal species. Metabolic pathways (integrated) were used as the pathway database; the hypergeometric test was used for algorithm selection for enrichment analysis, degree centrality as the topology measure, and combining *p*-values as the integration method (pathway-level).

Significantly enriched pathways were identified by comparing the effects of CS and HU, with an FDR of <0.05 and impact > 0.3, metabolite and protein hits of ≥2 in the pathway, and a pathway score of >2.0. The score was calculated as follows:

$$\begin{array}{l} Pathway\;score\\ =\;|median\;log_{2}\;FCmetHU\;-\;median\;log_{2}\;FCmetCS|\;\times \;log_{2}(hits\;met\;+\;1)\;\times \;log_{2}(hits\;pro+1)\;\times \;Impact \end{array}$$

The composite pathway score was defined as described above, integrating the effect size (delta median log_2_FC of metabolome in the pathway), molecular support (log2-transformed number of detected metabolites and proteins in the pathway), and pathway topology (Impact). This approach is conceptually consistent with topology-based pathway analysis methods, such as signaling pathway impact analysis, and with recently described multi-omics pathway scoring frameworks [23]. Binary comparisons, such as control (Ctrl) versus CS or Ctrl versus HU, were evaluated within the same pathways, which were significantly enriched in the CS-HU comparison. The total component, hit metabolite and protein numbers, raw *p*-value, −log_10_(*p*)-value, Holm-adjusted value, FDR, and Impact value for the significant pathways are presented in Supplementary Table S3.

Upward and downward trends were defined as the median log_2_FC of component metabolites and proteins in the pathway. 

Metabolites and proteins in the citrate cycle and pentose phosphate pathway (PPP) were evaluated using ANOVA with Tukey’s post hoc test, and statistical significance was defined as an FDR < 0.05.

## 3. Results

### 3.1. Evaluation of the Cytotoxicity of CS and HU

**Figure 1:**
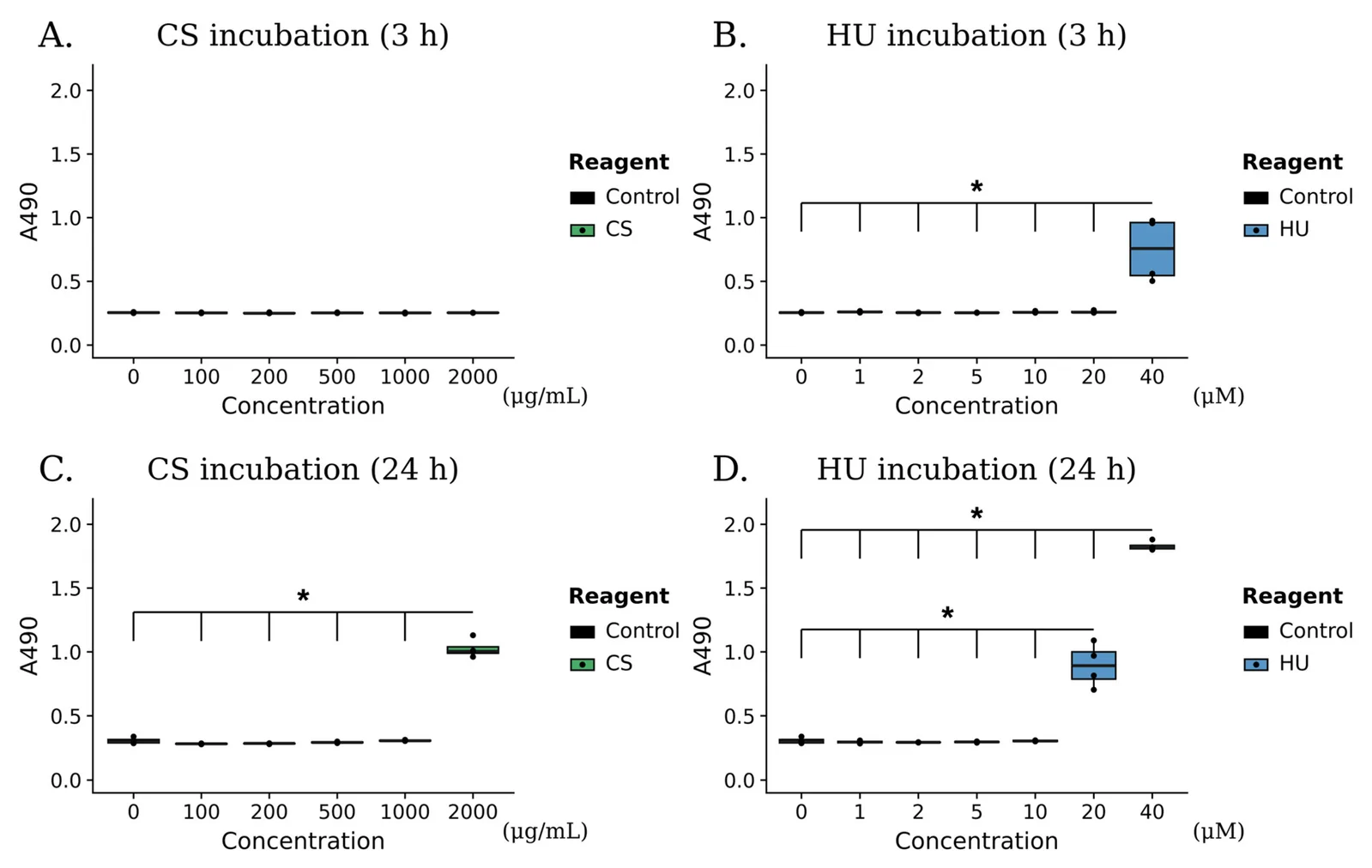
Cytotoxicity of CS and HU against GT1-7 cells after 3 and 24 h of incubation. GT1-7 cells were incubated with the indicated concentrations of CS or HU for 3 h and 24 h. Cells were exposed to 0, 100, 200, 500, 1000, or 2000 µg/mL of CS for 3 h (**A**) or 24 h (**C**). Cells were exposed to 0, 1, 2, 5, 10, 20, or 40 µM HU for 3 h (**B**) or 24 h (**D**). Cytotoxicity was expressed as the absorbance at 490 nm (A490). Significant differences are indicated by asterisks (FDR < 0.05; *n* = 4). Statistical significance was assessed using one-way ANOVA followed by Tukey’s post hoc test.

The cytotoxicity of CS and HU after 3 h and 24 h of exposure was evaluated using LDH assays in culture medium. Cytotoxicity was observed with 40 µM HU at 3 h, and with 2000 µg/mL CS and 20 and 40 µM HU at 24 h (Figure 1). For each substance, therefore, we selected the lowest concentration producing significant cytotoxicity at 24 h that was not cytotoxic at 3 h, and GT1-7 cells were incubated with 2000 µg/mL CS or 20 µM HU for 3 h in subsequent experiments. Quantification of cannabinoids in the exposure medium, after removal of the precipitate that formed on addition of the DMSO stock, showed that a nominal concentration of 2000 µg/mL CS delivered 166 nM THC, 800 nM THCA, and 11.3 nM CBD (Supplementary Table S4). Assuming complete decarboxylation of THCA, the combined THC and THCA concentration corresponds to 0.97 µM THC-equivalent—approximately one-twentieth of the 20 µM HU exposure in molar terms—and is delivered by a low-efficacy partial agonist rather than by a full agonist.

### 3.2. Multivariate Analysis of the Effect of CS and HU on Metabolome and Proteome Reprogramming in GT1-7 Cells

**Figure 2:**
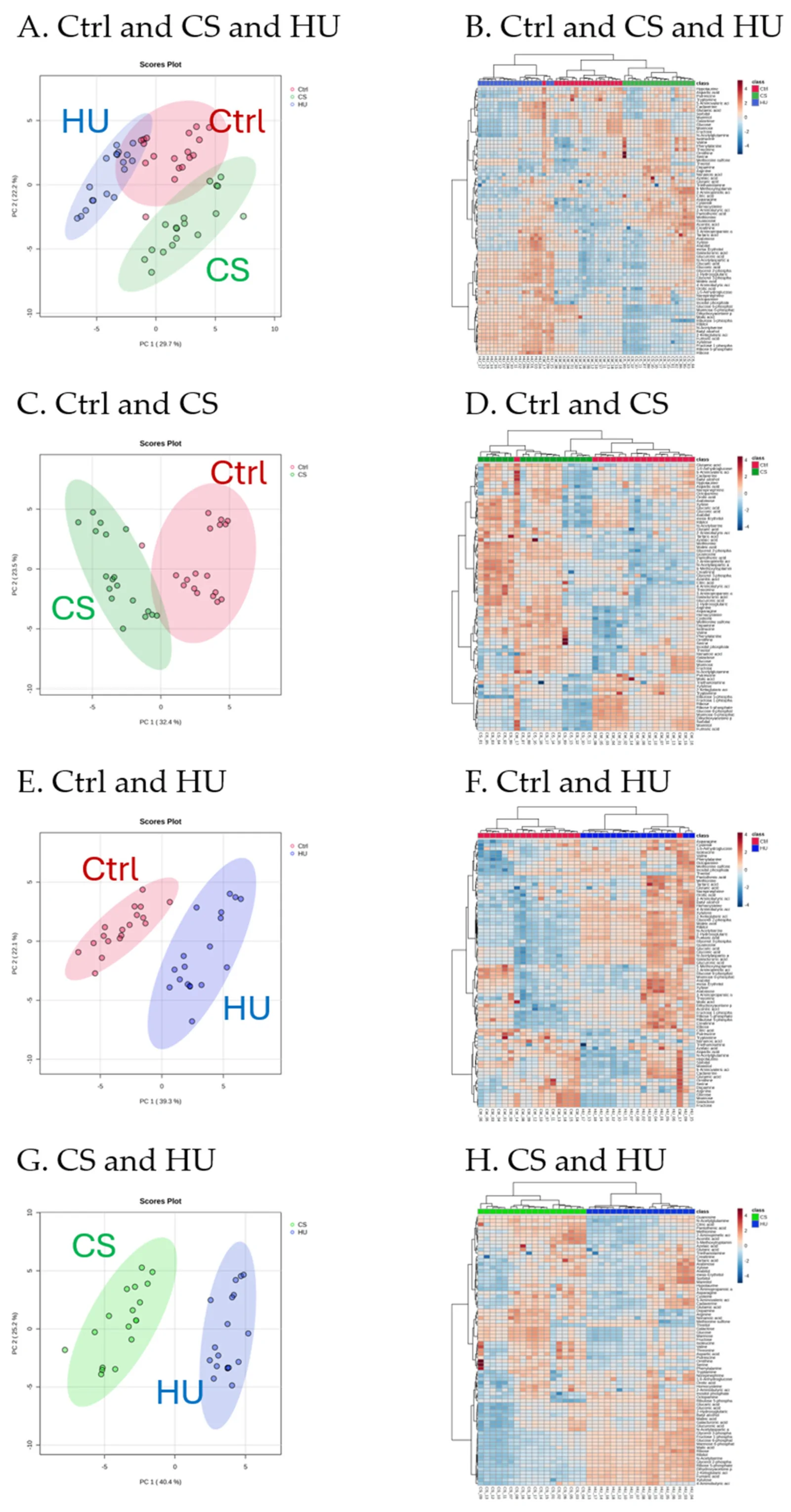
Multivariate and clustering analyses of the metabolome of GT1-7 cells after treatment with CS or HU for 3 h. Principal component analysis (PCA) score plot and heatmap of the 75 metabolites for each treatment in GT1-7 cells treated for 3 h are shown. PCA score plot for Ctrl, CS, and HU (**A**); PCA score plot for Ctrl and CS (**C**); PCA score plot for Ctrl and HU (**E**); PCA score plot for CS and HU (**G**). Heatmap for Ctrl, CS, and HU (**B**); heatmap for Ctrl and CS (**D**); heatmap for Ctrl and HU (**F**); heatmap for CS and HU (**H**); (*n* = 18).

After the metabolomic analysis, a multivariate analysis and hierarchical clustering of 75 metabolites (Supplementary Table S1) were performed. The PCA score plot and heatmap for the Ctrl, CS, and HU groups were well separated and clustered (Figure 2A,B). Binary comparisons (such as Ctrl versus CS, Ctrl versus HU, and CS versus HU) were also separated and clustered (Figure 2B–H). The significant metabolites identified in CS- and HU-treated cells compared with Ctrl cells are shown in Table 1 and Table 2, respectively. These results suggest that both CS and HU differentially affect metabolome reprogramming in GT1-7 cells after 3 h of exposure.

**Figure 3:**
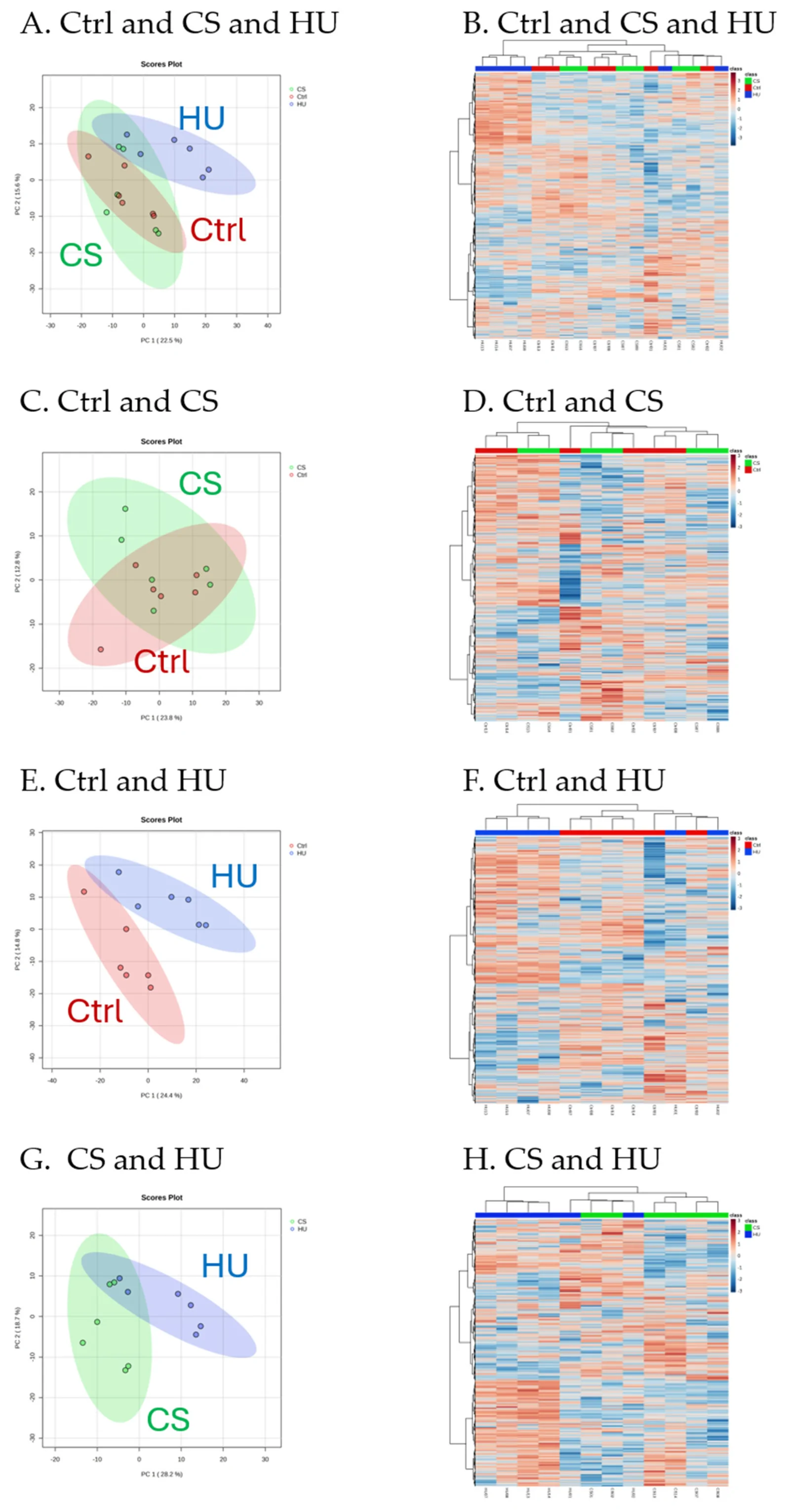
Multivariate and clustering analyses of the proteome of GT1-7 cells following treatment with CS or HU for 3 h. PCA score plot and heatmap for 2720 proteins for GT1-7 cells subjected to each treatment for 3 h are shown. PCA score plot for Ctrl, CS, and HU (**A**); PCA score plot for Ctrl and CS (**C**); PCA score plot for Ctrl and HU (**E**); PCA score plot for CS and HU (**G**). Heatmap for Ctrl, CS, and HU (**B**); heatmap for Ctrl and CS (**D**); heatmap for Ctrl and HU (**F**), heatmap for CS and HU (**H**); (*n* = 6).

Based on the analysis according to the workflows shown in Supplementary Figure S2, a total of 2720 proteins were subjected to multivariate analysis and hierarchical clustering following a proteome selection scheme (Supplementary Figure S2). The PCA score plot and heatmap for the three groups (Ctrl, CS, and HU) showed that the Ctrl and CS groups did not separate in either method; by contrast, the Ctrl and HU groups were separated (Figure 3A,B). In binary comparisons, the Ctrl and CS groups did not separate or cluster in the PCA score plot and heatmap, whereas the Ctrl and HU groups did (Figure 3C–F). A comparison of the CS and HU groups in the PCA score plot and heatmap showed almost complete separation and clustering (Figure 3G,H). These results indicate that HU produced substantially greater changes in the proteome than CS. Of the 267 proteins that differed significantly among the three groups, 186 were significantly changed in HU-treated cells relative to the control, whereas 8 were significantly changed in CS-treated cells; the latter did not yield pathway-level enrichment.

All 2720 proteins were screened for changes in expression induced by CS and HU using one-way ANOVA with Tukey’s post hoc test (alpha = 0.05). Statistical analysis revealed significant changes in the expression of 267 proteins (Supplementary Table S2). The statistically significant proteins identified in CS- and HU-treated cells compared with Ctrl cells are presented in Table 3 and Table 4, respectively. An integrated pathway analysis was conducted for 256 annotated proteins (of 267) and 75 metabolites using MetaboAnalyst, as described below. Two levels of statistical analysis are reported. Table 4 lists proteins that differed significantly between control and HU-treated cells in a screen of all 2720 quantified proteins, with false discovery rate control applied across that full set. 

### 3.3. Integrated Pathway Analysis Revealed CS and HU Exert Differing Effects on the Metabolome and Proteome

The results described above suggest that CS and HU exert differing effects on metabolomic and proteomic reprogramming in GT1-7 cells. HU is a pure chemical compound widely used as a CB1R agonist; by contrast, CS consists of multiple compounds, including terpenoids, flavonoids, and CBs such as THC, one of the most well-known CBs. This complexity enables a system-level analysis of metabolic regulation that cannot be captured using a single compound.

**Figure 4:**
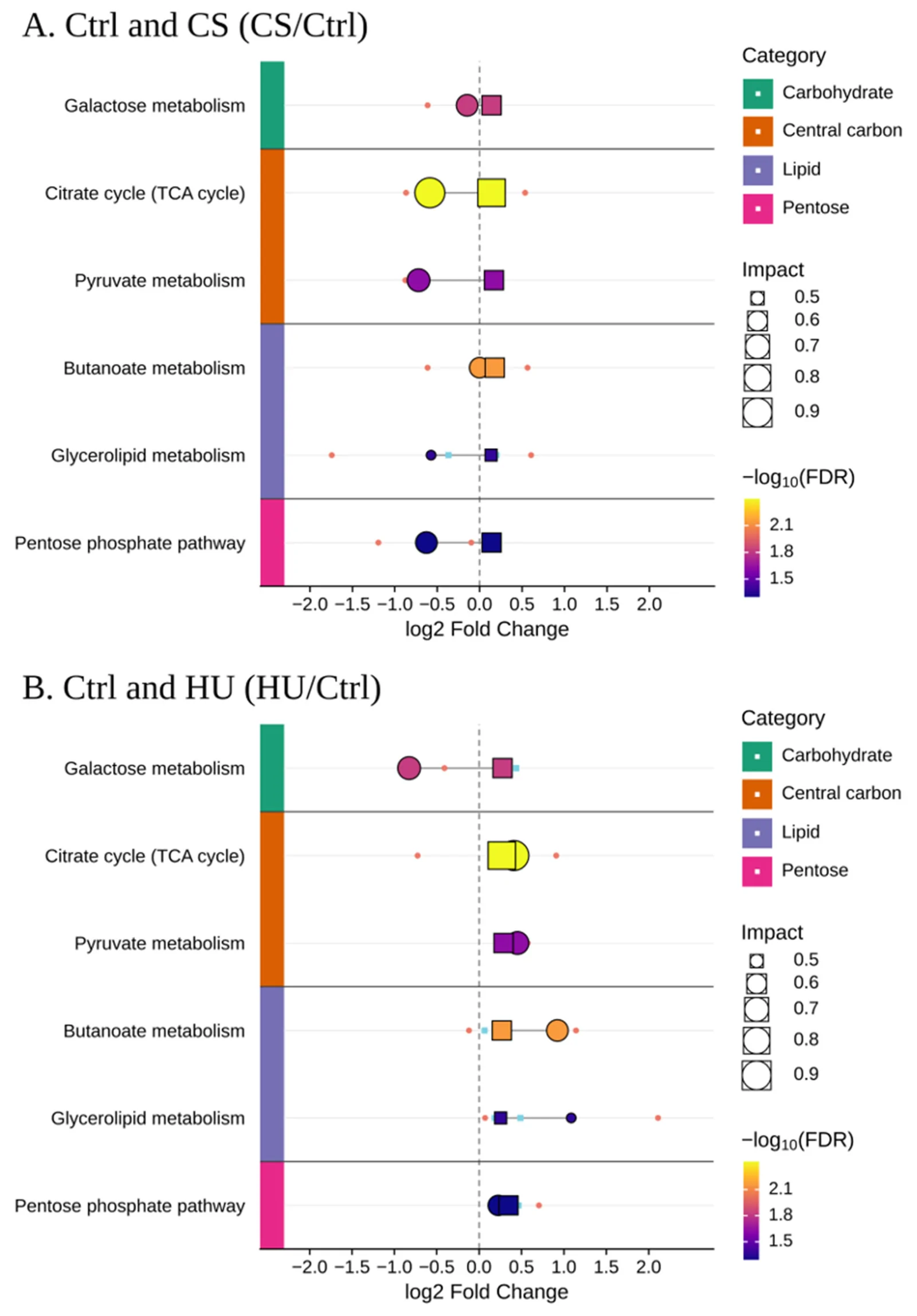
Pathway analysis integrating the metabolome and proteome of GT1-7 cells. Trends for each pathway are shown, integrating the metabolome (75 metabolites) and proteome (256 annotated proteins by joint enrichment analysis). Ctrl and CS (CS/Ctrl) (**A**); Ctrl and HU (HU/Ctrl) (**B**). Colored circles and squares represent the log2 median FC of the metabolome and the proteome annotated in the indicated pathway, respectively. Symbol size is correlated with impact size, and color shows the −log10 (FDR) for the pathway. Small orange circles and blue squares represent individual log2FC values for metabolites and proteins annotated in the pathway, respectively (metabolome: *n* = 18; proteome: *n* = 6). Proteome data symbols were plotted at +0.12 of the intact values to avoid overlap with the metabolome symbols shown.

A joint pathway analysis was conducted to elucidate differences in the effects of HU and CS on cells, focusing on upward and downward trends in metabolite production and protein expression within significant pathways. After analysis of the metabolome (75 metabolites) and proteome (256 proteins) and filtering according to pathway score criteria, as described in the Materials and Methods Section, six significantly altered pathways were identified: citrate cycle, pyruvate metabolism, glycerolipid metabolism, butanoate metabolism, PPP, and galactose metabolism (Figure 4). The metabolomic and proteomic trends across these pathways are shown in Figure 4. The significant pathways showed an upward trend in the metabolomes of HU-treated cells and a downward trend in CS-treated cells compared with the Ctrl, except for galactose and butanoate metabolism. Butanoate metabolism remained essentially stable in CS-treated cells, whereas galactose metabolism exhibited a downward trend in HU-treated cells and a relatively downward trend in CS-treated cells (Figure 4A,B). With regard to the proteome, the significant pathways in CS-treated cells were relatively stable, whereas an upward trend was noted in HU-treated cells (Figure 4).

**Figure 5:**
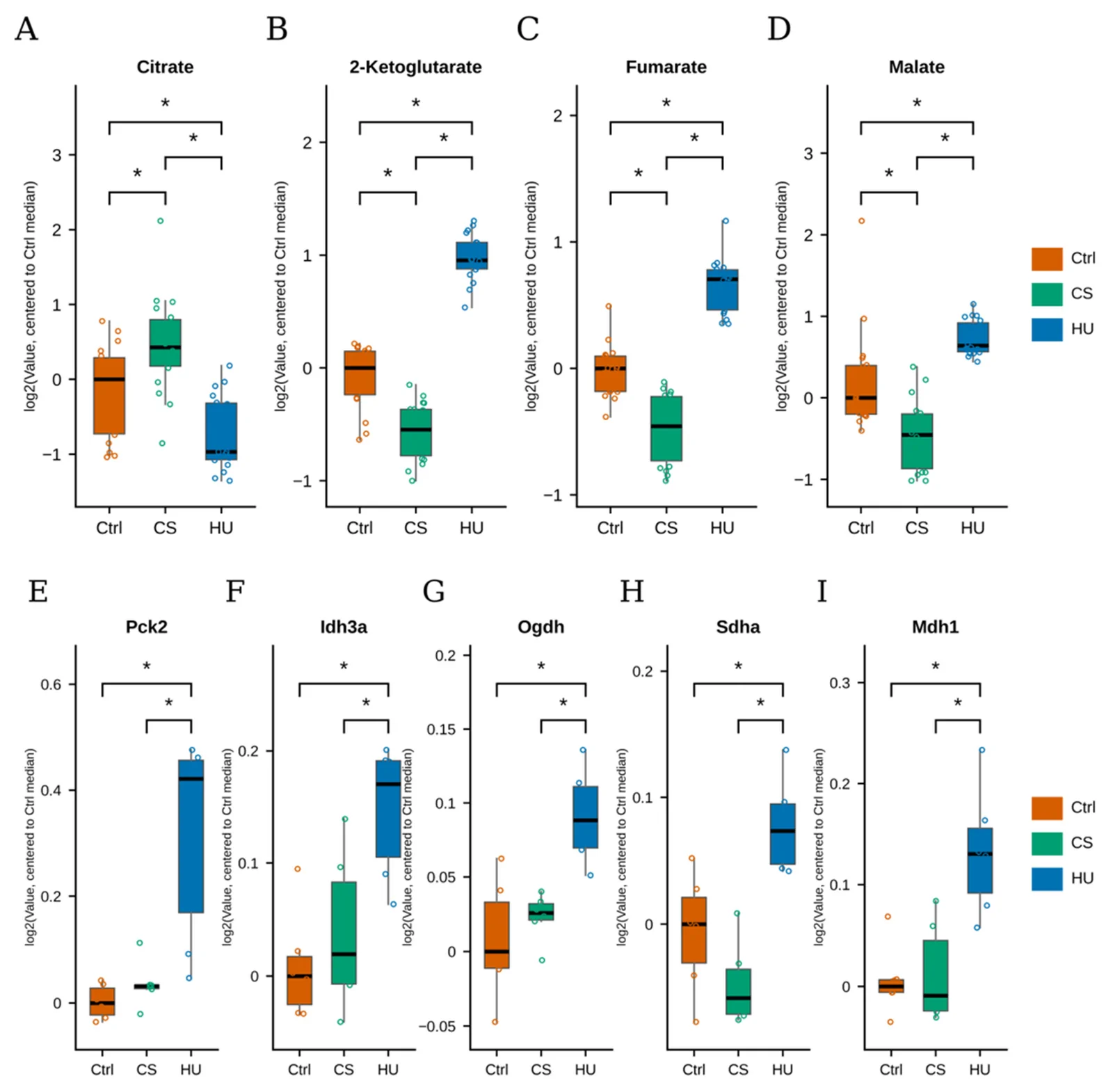
Metabolites and proteins involved in the citrate cycle in GT1-7 cells. Metabolites and proteins associated with the TCA cycle are shown. Metabolites include citrate (**A**), 2-ketoglutarate (**B**), fumarate (**C**), and malate (**D**). Proteins include Pck2 (**E**), Idh3a (**F**), Ogdh (**G**), Sdha (**H**), and Mdh1 (**I**). Significant differences between treatments are indicated with asterisks (FDR < 0.05; metabolites: *n* = 18; proteins: *n* = 6). Statistical significance was assessed using one-way ANOVA followed by Tukey’s post hoc test.

The citrate cycle, which functions between glycolysis in the cytosol and oxidative phosphorylation in the mitochondria, plays a major role in energy production and providing substrates for other pathways. The PPP provides substrates for glycolysis and nucleic acid production and plays an important role in antioxidant defense via the production of NADPH. These pathways play central roles in the maintenance of biological homeostasis, and Figure 5 shows the associated metabolites and proteins detected in this study.

Citrate cycle metabolites differentially affected by CS and HU included citrate, 2-ketoglutarate, fumarate, and malate. Citrate levels were increased in CS-treated cells and decreased in HU-treated cells compared with Ctrl cells (Figure 5A). Conversely, 2-ketoglutarate, fumarate, and malate levels were significantly decreased in CS-treated cells and increased in HU-treated cells compared with Ctrl cells, with significant differences also observed between CS- and HU-treated cells (Figure 5B–E). Five differentially expressed proteins were identified in the same pathway: phosphoenolpyruvate carboxykinase (Pck2), isocitrate dehydrogenase subunit α (Idh3a), 2-oxoglutarate dehydrogenase complex component E1 (Ogdh), succinate dehydrogenase flavoprotein subunit (Sdha), and malate dehydrogenase (Mdh1). All these proteins were upregulated in HU-treated cells; however, no significant differences in expression were observed in CS-treated cells (Figure 5E–I).

**Figure 6:**
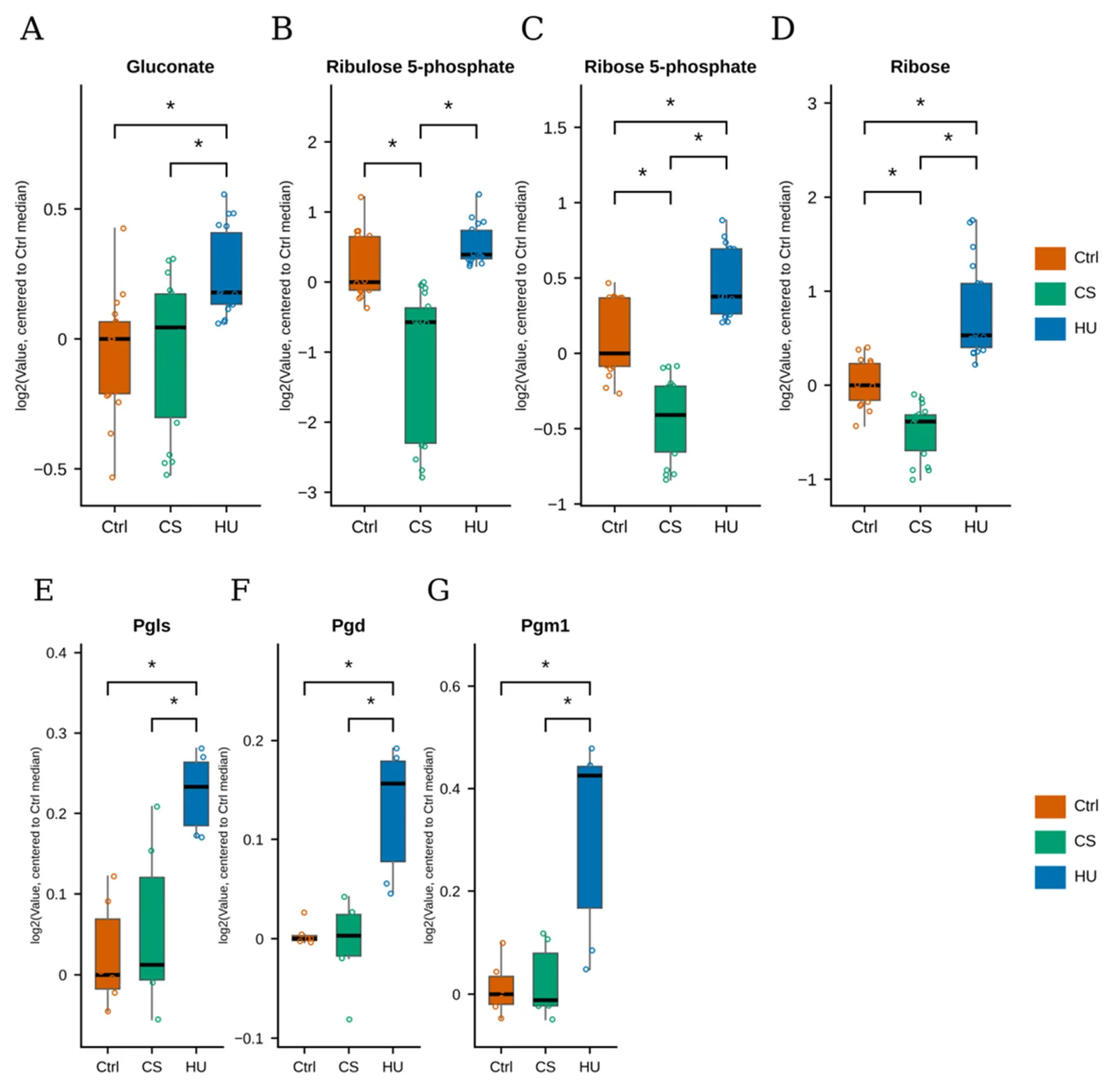
Metabolites and proteins involved in the pentose phosphate pathway in GT1-7 cells. Metabolites and proteins annotated in the pentose phosphate pathway are shown. Metabolites include gluconate (**A**), ribulose-5-phosphate (**B**), ribose-5-phosphate (**C**), and ribose (**D**). Proteins include Pgls (**E**), Pgd (**F**), and Pgm1 (**G**). Significant differences between treatments are indicated with asterisks (FDR < 0.05; metabolites: *n* = 18; proteins: *n* = 6). Statistical significance was assessed using one-way ANOVA followed by Tukey’s post hoc test.

Three differentially expressed PPP proteins were identified: 6-phosphogluconolactonase (Pgls), 6-phosphogluconate dehydrogenase (Pgd), and phosphoglucomutase-1 (Pgm1). All of them were upregulated in HU-treated cells compared with Ctrl and CS-treated cells, but no significant differences were observed in CS-treated cells relative to Ctrl cells (Figure 6E–G).

Collectively, these results suggest that HU upregulates the citrate cycle and PPP in GT1-7 cells. The expression of proteins associated with these pathways was essentially unchanged in CS-treated cells, and levels of substrates other than citrate were decreased in both pathways.

Four PPP metabolites were significantly enriched (Figure 6). Gluconate, ribose-5-phosphate, and ribose levels were significantly increased in HU-treated cells compared with Ctrl and CS-treated cells (Figure 6A,C,D). Ribulose-5-phosphate was the only metabolite for which levels were not significantly altered in HU-treated cells compared with Ctrl cells; however, this metabolite was increased in HU-treated cells compared with CS-treated cells (Figure 6B). Gluconate did not change relative to the Ctrl in CS-treated cells; however, levels of ribulose-5-phosphate, ribose-5-phosphate, and ribose were decreased in CS-treated cells compared with both Ctrl and HU-treated cells (Figure 6A–D).

## 4. Discussion

### 4.1. Principal Findings

In this study, we performed integrated metabolomic and proteomic analyses of GT1-7 mouse hypothalamic neurons treated with either CS or HU for 3 h, a time point at which neither compound induced detectable cell death, whereas both compounds induced comparable levels of cell death after 24 h of exposure. These analyses revealed changes in opposite directions between the two exposures in the steady-state levels of metabolites across the citrate cycle, PPP, pyruvate metabolism, galactose metabolism, glycerolipid metabolism, and butanoate metabolism, although metabolic flux was not measured. At the protein level, upregulation was observed exclusively in HU-treated cells. These findings indicate that CS and HU elicit qualitatively distinct early-phase metabolic responses during progression toward the common endpoint of cell death. THC, the principal psychoactive constituent of Cannabis, has been shown to induce apoptosis in cultured neurons, characterized by nuclear condensation, DNA fragmentation, arachidonic acid release, and oxidative stress-dependent cell death [24,25]. This apoptosis response can be attenuated by antioxidants such as vitamin E or cyclooxygenase inhibitors such as aspirin, suggesting that reactive oxygen species (ROS) production is the primary mechanism of cell death [25]. However, Cannabis is not a single-component substance but rather a complex mixture including other CBs, terpenes, and flavonoids. The combined actions of these constituents determine the toxicity profile of Cannabis extracts [24].

The neurotoxicity of synthetic CBs against neuronal cells was characterized in detail by Tomiyama and Funada using primary mouse forebrain neurons [26]. Their study demonstrated that HU and several other synthetic CBs induce cell death via a CB1R-dependent mechanism, with no involvement of CB2R. Using the representative compound CP-55,940, annexin V-positive cells and activated caspase-3 were detected, which established CB1R-mediated, caspase-3-dependent apoptosis as the neurotoxic effect. HU is a full agonist at CB1R and CB2R. In other neuronal systems, HU has been reported to engage PI3K/Akt signaling associated with neuroprotection [14], although neither receptor dependence nor PI3K/Akt activation was examined in the present study. Both exposures used here are cytotoxic at 24 h. The 3 h time point therefore represents a pre-lethal state under a lethal exposure. Clear metabolic changes were nevertheless already present at 3 h, and these changes differed in direction between CS and HU, indicating that exposure-specific metabolic responses precede detectable cytotoxicity.

### 4.2. Comparability of the CS and HU Exposures

CS is a multi-component botanical preparation and HU is a single synthetic full agonist, and the two cannot be matched on a molar basis in any meaningful way. We therefore used an iso-effective design, selecting for each substance the lowest concentration producing significant LDH release at 24 h that was non-cytotoxic at 3 h (Figure 1), so that the two exposures are matched on the toxicological endpoint of interest rather than on chemical concentration. The same medium preparation procedure was used for the cytotoxicity assays and for the omics experiments, so that Figure 1 and the omics data refer to identical exposure conditions.

At a nominal concentration of 2000 µg/mL, CS delivered 166 nM THC, 800 nM THCA, and 11.3 nM CBD, corresponding to 0.97 µM THC-equivalent if THCA is assumed to decarboxylate completely. These concentrations are low, for reasons that we can identify: chloroform extraction may have recovered only a hydrophobic fraction of the cannabinoid content. The delivered concentrations therefore reflect the soluble fraction of the preparation rather than the nominal dose.

Three consequences follow for the interpretation of our results. First, the cannabinoid exposure delivered by CS was approximately twentyfold lower in molar terms than the 20 µM HU exposure and was delivered by a low-efficacy partial agonist rather than by a full agonist with sub-nanomolar CB1R affinity [11,12]; the difference in cannabinoid receptor engagement between the two exposures is therefore likely to span several orders of magnitude. Second, and consequently, the cytotoxicity of CS at 24 h is unlikely to be attributable to THC, since concentrations reported to be cytotoxic to neuronal cells in vitro are substantially higher than those delivered here. The constituents responsible for the CS response were not identified in this study. Third, this asymmetry means that the divergent metabolic responses we observed should not be read as a comparison between two cannabinoid receptor agonists at matched receptor occupancy. They are a comparison between a defined, saturating exposure to a synthetic full agonist and a complex botanical exposure in which cannabinoids are present at sub-micromolar concentrations.

### 4.3. Divergent Metabolite Patterns in the Citrate Cycle and Pentose Phosphate Pathway

The citrate cycle is initiated by citrate synthase-catalyzed condensation of acetyl-CoA and oxaloacetate to form citrate, followed by aconitase (Aco2)-mediated isomerization to isocitrate, NAD^+^-dependent oxidative decarboxylation of isocitrate to 2-ketoglutarate by Idh3, Ogdh (2-oxoglutarate dehydrogenase complex)-catalyzed production of succinyl-CoA, Sdha-mediated formation of fumarate, and fumarase-catalyzed hydration to malate [27].

In HU-treated cells, citrate decreased while downstream metabolites (2-ketoglutarate, fumarate, and malate) increased. An increase in the steady-state level of a metabolite is ambiguous, since it may reflect either increased production or reduced downstream consumption. Here, however, the divergent direction of citrate and its downstream products is difficult to explain by downstream blockade alone and is compatible with—though not demonstrative of—increased throughput at the Aco2-catalyzed step and beyond. Citrate accumulation typically results from inhibition of Aco2, particularly oxidative inactivation of the iron–sulfur cluster under conditions of oxidative stress, or from inhibition of Idh3 under conditions of high NADH/ATP levels [27,28]. Although citrate was decreased in HU-treated cells, the proteomic analysis revealed no change in Aco2 levels, suggesting that citrate was converted to isocitrate via Aco2 and that the isocitrate produced was oxidatively decarboxylated to 2-ketoglutarate via Idh3a. Idh3a catalyzes the principal NAD^+^-to-NADH oxidoreduction step of the citrate cycle. Its abundance was increased in HU-treated cells relative to both control and CS-treated cells, although the magnitude was modest (×1.10 versus control). Whether this translates into an altered rate of NADH supply to the electron transport chain cannot be determined from protein abundance alone, and NADH/NAD^+^ was not measured.

Considerable interest has focused on 2-ketoglutarate under conditions of oxidative stress, not only because of its capacity for scavenging non-enzymatic H_2_O_2_ but also because of its reported ability to suppress neuronal senescence by inhibiting the mTOR pathway [29,30]. In addition, 2-ketoglutarate functions as a co-substrate for 2-oxoglutarate-dependent dioxygenases and therefore plays a role in epigenetic regulation through control of DNA and histone methylation [30].

Increased expression of Ogdh and Sdha was also detected in HU-treated cells, in each case relative to both control and CS-treated cells. Ogdh, the principal catalytic subunit of the 2-oxoglutarate dehydrogenase complex, mediates the conversion of 2-ketoglutarate to succinyl-CoA, with concomitant NADH production. Sdha, a flavoprotein subunit of succinate dehydrogenase (complex II), catalyzes the oxidation of succinate to fumarate. The increase in Mdh1 (malate dehydrogenase 1) abundance observed in the present study was distinct from that of the mitochondrial counterpart Mdh2, as Mdh1 is a cytoplasmic enzyme. In the malate–aspartate shuttle (MAS), Mdh1 catalyzes the reduction of oxaloacetate to malate in the cytoplasm while regenerating NAD^+^ from NADH. The MAS is the most critical shuttle mechanism in neurons for indirectly transferring cytoplasmic reducing equivalents into mitochondria to support oxidative phosphorylation [31,32]. Mdh1 abundance increased modestly in HU-treated cells (×1.09), which may indicate an altered capacity to transfer cytosolic reducing equivalents to mitochondria, but shuttle activity was not measured. We do not draw any conclusion regarding the overall efficiency of energy metabolism, which our data do not address. Pck2 catalyzes the conversion of mitochondrial oxaloacetate to phosphoenolpyruvate, linking the citrate cycle intermediate pool to the glycolytic pool in a cataplerotic reaction; its abundance increased in HU-treated cells (×1.14).

In CS-treated cells, citrate was increased relative to control and HU-treated cells, while 2-ketoglutarate, fumarate, and malate were decreased relative to both (Figure 5), the opposite of the pattern seen with HU. This profile is compatible with either a relative increase in citrate production or an impairment of its subsequent oxidation. Inhibition of mitochondrial respiratory complexes by THC has been reported by Wolff et al. [33], but the THC concentration delivered here (166 nM) is far below those used in their work, and we therefore do not attribute the CS response to this mechanism.

Thus, the CS-associated accumulation of citrate and decrease in downstream metabolites may indicate a reduction in citrate cycle turnover for energy generation. Notably, none of the citrate cycle enzymes quantified here differed significantly between CS-treated and control cells, even though the corresponding metabolites did. The metabolite changes produced by CS at 3 h were therefore not accompanied by changes in the abundance of the enzymes of the same pathway, in contrast to HU-treated cells, in which enzymes and metabolites changed concordantly.

The oxidative PPP comprises three sequential reactions: glucose-6-phosphate (G6P) → 6-phosphogluconolactone (catalyzed by G6PD), → 6-phosphogluconate (catalyzed by Pgls), and → ribulose-5-phosphate + NADPH (catalyzed by Pgd). Pgls (6-phosphogluconolactonase) rapidly hydrolyzes 6-phosphogluconolactone to 6-phosphogluconate, preventing accumulation of the isomerized (γ-form) product and thereby ensuring unimpeded progression through the PPP [34]. The reaction catalyzed by Pgd generates the second molecule of NADPH while producing ribulose-5-phosphate, making it one of the two major NADPH-generating steps of the oxidative PPP [34,35]. In HU-treated cells, Pgls, Pgd, and Pgm1 abundance was increased relative to control and CS-treated cells, and gluconate, ribose-5-phosphate, and ribose were likewise increased relative to both (Figure 6); ribulose-5-phosphate was increased relative to CS-treated cells. Enzyme and metabolite changes within this pathway were therefore concordant in direction. However, all measured intermediates changed in the same direction, so increased oxidative throughput cannot be distinguished from accumulation due to reduced downstream consumption, and NADPH was not measured in this study. Ribulose-5-phosphate is further converted via the non-oxidative PPP to ribose-5-phosphate and ribose, which serve as precursors for nucleotide synthesis. Ribose and ribose-5-phosphate levels were increased in HU-treated cells; whether this reflects increased production or reduced consumption—for example, accumulation upstream of a block in nucleotide biosynthesis—cannot be determined from these data, and no inference regarding nucleotide synthesis capacity is warranted. Pgm1, which catalyzes the interconversion of glucose-1-phosphate and G6P at a node linking glycogen metabolism, glycolysis, and the PPP, was likewise increased in HU-treated cells relative to control and CS-treated cells. This is compatible with a redistribution of glucose-6-phosphate toward the PPP, although pathway partitioning was not measured.

In CS-treated cells, ribulose-5-phosphate, ribose-5-phosphate, and ribose were decreased relative to control and HU-treated cells, and gluconate was decreased relative to HU-treated cells (Figure 6). No PPP enzyme differed significantly from the control in CS-treated cells. Whether the reduced intermediate levels reflect decreased production or increased consumption cannot be determined from these data.

Galactose is taken up by neurons via glucose transporter 3 and channeled through the Leloir pathway (galactose → glucose-1-phosphate → G6P) to supply carbon for glycolysis and the PPP [36]. Compensatory upregulation of galactose metabolism under conditions of glucose deficiency has been reported [37], which reflects the capacity of neurons to switch flexibly between energy substrates. Galactose metabolism showed no clear change in CS-treated cells, indicating that the broad suppression of central carbon metabolism observed with CS was not accompanied by compensatory recruitment of an alternative carbon source. In HU-treated cells, galactose metabolism showed a downward trend. One possible interpretation is a reduced relative contribution of galactose-derived carbon.

Butanoate (butyrate) metabolism is linked to the entry of GABA into the citrate cycle via the GABA shunt (GABA → succinic semialdehyde → succinate) [38]. Butanoate metabolism showed an upward trend in HU-treated cells and was essentially unchanged in CS-treated cells. GABA was not among the metabolites detected, and the GABA shunt was not assessed.

Within glycerolipid metabolism, only two metabolites were detected: dihydroxyacetone phosphate and glycerol-3-phosphate. Both were increased in HU-treated cells and decreased in CS-treated cells. Because both lie at the branch point between glycolysis and glycerolipid synthesis, these changes may reflect altered availability of glycolytic intermediates rather than a response specific to glycerolipid metabolism.

In CS-treated cells, both metabolites were reduced. One possible contributor is reduced availability of dihydroxyacetone phosphate, the glycolytic precursor of glycerol-3-phosphate, consistent with the broad suppression of central carbon metabolism observed in these cells. A receptor-independent suppression of arachidonoyl acylation of phosphatidylinositol and triacylglycerol by THC has also been reported [39], but this effect was observed at concentrations far exceeding those delivered here, and we do not suggest it as an explanation. Because protein levels were essentially unchanged, changes in metabolite and substrate availability, rather than altered abundance of lipid-metabolizing enzymes, are the more likely proximate basis for these differences.

### 4.4. Proteome Responses

Although the CS proteome was largely unchanged, HU treatment was associated with increased abundance of citrate cycle-related enzymes (Idh3a, Ogdh, Sdha, and Mdh1), Pck2, and PPP-related enzymes (Pgls, Pgd, and Pgm1). The magnitude of the individual protein changes was modest (typically 5–20%; median |log_2_FC| = 0.17 across the 186 proteins significantly changed by HU). We therefore do not interpret any single protein change as functionally decisive; the informative observation is that multiple enzymes within the same pathway changed concordantly and in the same direction as the corresponding metabolites. Consistent with an oxidative-stress component, peroxiredoxin-6 (Prdx6, ×1.13), peroxiredoxin-2 (Prdx2, ×1.09), and thioredoxin domain-containing protein 5 (Txncd5, ×1.17) were also increased in HU-treated cells, although no functional redox measurement was performed. In CS-treated cells, only 8 of 2720 proteins changed significantly, and these did not produce pathway-level enrichment. Two of the eight were ribosomal proteins; large ribosomal subunit protein eL29 (Rpl29, ×0.63), ubiquitin-like FUBI-ribosomal protein eS30 fusion protein (Fau, ×0.67), and sequestosome-1 (Sqstm1, ×0.78) were also decreased. Metabolite changes were therefore extensive at 3 h, while proteome changes remained minimal. Differences between CS and HU in CB1R affinity and in other molecular targets, and the resulting differences in the intensity and specificity of downstream signaling, may contribute to the qualitative divergence in cellular responses.

### 4.5. Mechanistic Hypotheses Arising from These Data

The results of these analyses indicate that CS and HU elicit opposing metabolic responses at the early 3 h time point. They are presented as hypotheses generated by the observed metabolite and protein patterns, to be tested in future work, and should not be read as mechanisms established by the present data.

Proteomic studies have shown that CB1R activation in hippocampal neurons upregulates the expression of ribosomal proteins and translation initiation factors, indicative of enhanced protein synthesis [40], and PI3K/Akt/mTOR signaling has been proposed to drive such translational changes [4].

Pck2 expression has been reported to be induced through the GCN2–eIF2α–ATF4 axis under amino acid deprivation and endoplasmic reticulum stress, and to contribute to maintenance of the reduced glutathione pool [41,42].

NADPH serves as the electron donor for glutathione reductase-mediated regeneration of reduced glutathione, and PPP activation with maintenance of the NADPH/NADP^+^ ratio has been reported to underlie the neuroprotective effect of CBD in hippocampal ischemia–reperfusion injury [43].

CB1R is densely expressed at GABAergic synapses, where it has a central role in regulating inhibitory neurotransmission. Modulation of GABAergic metabolism therefore represents one hypothesis for the increase in butanoate metabolism. In other systems, mTORC1–SREBP-1 signaling promotes lipogenic gene expression [44], and DAG serves as the substrate for DAG lipase α-mediated 2-AG synthesis [45].

The pattern of changes observed in HU-treated cells generates the following hypotheses, none of which was tested in this study and each of which requires direct experimental examination: (i) altered protein synthesis via PI3K/Akt/mTOR signaling [14,40]; (ii) altered citrate cycle throughput associated with the coordinated increases in Idh3a, Ogdh, and Sdha abundance [29,30]; (iii) altered malate–aspartate shuttle capacity associated with increased Mdh1 abundance [31,32]; (iv) altered oxidative PPP throughput and NADPH production associated with the coordinated increases in Pgls, Pgd, and Pgm1 abundance [34,35,43]; (v) Pck2-mediated modulation of the link between the citrate cycle and glycolysis [41,42]; (vi) altered glycerolipid synthesis via the mTORC1–SREBP-1 axis [14,44]. We emphasize that these are inferences drawn from abundance data in conjunction with the literature, and that the individual protein changes were modest in magnitude.

### 4.6. Convergent Lethality via Divergent Early Metabolic Trajectories

Because both 2000 µg/mL CS and 20 µM HU are cytotoxic at 24 h, the 3 h time point examined here represents a pre-lethal state under a lethal exposure. We therefore interpret the observed changes as early, exposure-specific stress responses of undetermined functional consequence.

Two interpretations remain equally consistent with our data. The coordinated increases in citrate cycle and PPP metabolites and enzymes in HU-treated cells may represent a compensatory response that was ultimately insufficient to prevent cell death. Alternatively, they may reflect a metabolic over-activation that itself contributed to injury—for example, through increased mitochondrial electron flow and consequent reactive oxygen species generation. Our data cannot distinguish between these possibilities, and we do not favor either. Sustained CB1R activation has been reported to suppress the cAMP/PKA/complex I axis through mitochondrial CB1R, reducing ATP production [46].

What our data do establish is a divergence of trajectory. CS produced broad metabolic suppression: citrate accumulated, downstream citrate cycle and PPP intermediates decreased, and the proteome was largely unchanged. HU produced the opposite pattern: citrate decreased, downstream intermediates increased, and multiple enzymes of the same pathways increased concordantly, albeit modestly. Both exposures converged on comparable cytotoxicity at 24 h. Metabolic arrest and metabolic over-activation are both compatible with cell death, and the finding we wish to emphasize is not that either response was protective, but that two exposures producing similar cytotoxic outcomes do so through early metabolic changes in opposite directions.

Because the quantified cannabinoids were delivered at sub-micromolar concentrations, the cytotoxicity of CS at 24 h is unlikely to be attributable to THC. Other constituents of the preparation—including cannabinoids that were not quantified, terpenes, flavonoids, and compounds outside these classes—were not characterized, and the agents responsible for the CS response therefore remain unidentified.

The present study employed a sequential metabolite and protein extraction method to enable integrated omics analyses across the metabolite and protein molecular layers in the same samples. Using this approach, we found that HU is toxic to GT1-7 cells and that its early metabolic signature differs in direction from that of CS. Synthetic CBs, including HU, are reportedly toxic to primary forebrain cells via a CB1R- and caspase-3-dependent mechanism [26]. This study is the first to characterize these early metabolic and proteomic responses using an integrated multi-omics approach. Nabiximols, a Cannabis extract containing an equal ratio of THC to CBD, is used medically to treat symptoms of multiple sclerosis [47,48] and is reportedly effective for ameliorating neuropathic pain [49,50]. These findings may inform future mechanistic studies of cannabinoid neurotoxicity.

### 4.7. Limitations

The involvement of CB1R in HU- and CS-induced cytotoxicity and metabolomic and proteomic remodeling in GT1-7 cells could not be directly assessed using AM251, a CB1R antagonist. The concentrations reportedly necessary to suppress synthetic CBs (including HU) induce neuronal death (>20 µM [26]), exceeding the cytotoxic threshold for GT1-7 cells in our system (>2 µM). CB1R involvement therefore remains untested, and all statements regarding receptor dependence in this manuscript should be read as hypotheses. Future work will address this using antagonist exposure restricted to sub-cytotoxic concentrations with shortened treatment intervals, rimonabant as an alternative antagonist, Cnr1 knockdown, and comparison with a CB2R-selective agonist.

In the present study, comprehensive analyses were performed using metabolomic and proteomic approaches. Because these analytical methods are not designed to target specific components, not all metabolites and proteins associated with the metabolic pathways modulated by HU or CS could be detected. The proteome served as a basis for the pathway-level analysis. In GT1-7 cells, which are characterized by reduced genetic and environmental variability, a total of six replicates could be considered sufficient, because many previous in vitro studies have adopted sextuplicate experiments. Future studies should attempt to validate these findings with larger sample sizes. Furthermore, the proteomic analysis conducted here did not account for post-translational modifications, such as phosphorylation, and was therefore limited to assessing protein expression levels. As such, changes in protein activity mediated by post-translational modifications within the affected pathways are not reflected in the present proteomic data. Nevertheless, it is likely that proteins for which expression was significantly altered by each treatment exert substantial impacts on the associated pathways.

Metabolite and protein abundances were measured; metabolic flux was not. Stable-isotope tracing is required to determine whether the observed changes reflect altered pathway throughput rather than altered pool size.

The signaling and redox variables mentioned in the interpretation of these data were not measured. AKT and mTOR phosphorylation, SREBP-1 nuclear translocation, NADPH/NADP^+^ and glutathione/glutathione disulfide ratios, reactive oxygen species, mitochondrial membrane potential, and 2-AG and DAG levels all remain to be determined.

Only a single early time point (3 h) and a single immortalized cell line were examined. Intermediate time points and primary neuronal cultures will be required to establish how the divergent early responses described here relate to the comparable cytotoxicity observed at 24 h.

Only cannabinoids were quantified in the CS preparation; terpenes, flavonoids and other constituents were not characterized. Because the quantified cannabinoids were contained at sub-micromolar concentrations, the constituents responsible for the CS response remain unidentified. In addition, because the two exposures were matched on cytotoxic effect rather than on concentration, we cannot separate concentration-dependent effects from effects attributable to compound class.

The magnitude of individual protein changes was modest (typically 5–20%; median |log_2_FC| = 0.17 across the 186 proteins significantly changed by HU). We therefore do not interpret any single protein change as functionally decisive.

Neither CB1R nor CB2R was detected in our proteomic dataset. This is expected rather than informative: G protein-coupled receptors are low-abundance integral membrane proteins that are poorly represented in data-dependent shotgun proteomics performed without membrane enrichment, and non-detection here does not indicate absence of expression. Expression of both receptors in GT1-7 cells has been reported previously [21,22]. Because we could neither detect the receptors nor test their involvement pharmacologically, we have avoided attributing any of the observed responses to a specific receptor.

The cannabinoid concentrations delivered by CS were low relative to the nominal exposure concentration, reflecting the age of the plant material and possible incomplete recovery during chloroform extraction. The exposure therefore represents the soluble fraction of the preparation rather than its total composition, and constituents with low aqueous solubility are likely to be under-represented relative to the plant material of origin.

Conversely, the 20 µM HU concentration used here exceeds the reported CB1R dissociation constant of this compound by several orders of magnitude [11]. Although this concentration was selected because of its cytotoxicity in this system, receptor-independent actions at such concentrations cannot be excluded.

## 5. Conclusions

Using sequential extraction of metabolites and proteins from a single cell pellet, we compared the early responses of GT1-7 neuronal cells to CS and HU at exposure levels matched for cytotoxicity at 24 h. At 3 h, a time point preceding detectable cell death, the two exposures altered the same pathways in opposite directions. The extract suppressed broad central carbon metabolism with an essentially unchanged proteome, whereas HU produced coordinated increases in citrate cycle and PPP metabolites, together with increases in the corresponding enzymes. Both exposures resulted in comparable cytotoxicity at 24 h. These results suggest that CS and HU, despite having similar cytotoxic endpoints, can be distinguished at the metabolome and proteome levels before this endpoint is reached.

## Figures and Tables

**Table 1:** List of significant metabolites identified in *Cannabis* extract-treated cells compared with the control group (n = 42).

Metabolites	t-Stat	*p*-Value	−log_10_(p)	FDR	Fold Change	log_2_(FC)
Guanosine	−28.871	1.68 × 10^−25^	24.775	1.26 × 10^−23^	18.40	4.20
Pantothenate	−16.337	1.15 × 10^−17^	16.94	2.87 × 10^−16^	2.21	1.15
Aconitate	−6.3964	2.64 × 10^−7^	6.5776	1.84 × 10^−6^	1.94	0.96
Creatinine	−3.0166	4.81 × 10^−3^	2.3175	9.26 × 10^−3^	1.75	0.80
Glycerol 2-phosphate	−5.7221	1.98 × 10^−6^	5.7038	9.27 × 10^−6^	1.73	0.79
Glycerol 3-phosphate	−4.7663	3.44 × 10^−5^	4.4629	9.94 × 10^−5^	1.65	0.72
4-Aminobutyrate	−6.2288	4.35 × 10^−7^	6.3613	2.51 × 10^−6^	1.61	0.69
Citrate	−2.9657	5.49 × 10^−3^	2.2604	1.03 × 10^−2^	1.57	0.65
Asparagine	−6.3597	2.95 × 10^−7^	6.5303	1.84 × 10^−6^	1.55	0.63
Phenylalanine	−4.8244	2.90 × 10^−5^	4.5379	9.06 × 10^−5^	1.48	0.57
2-Aminopimelic acid	−4.9604	1.93 × 10^−5^	4.7137	6.75 × 10^−5^	1.48	0.56
Methionine sulfone	−4.7838	3.27 × 10^−5^	4.4855	9.81 × 10^−5^	1.46	0.55
Maleate	−6.6009	1.44 × 10^−7^	6.8405	1.20 × 10^−6^	1.44	0.53
Isoleucine	−4.9522	1.98 × 10^−5^	4.7031	6.75 × 10^−5^	1.41	0.50
Methionine	−6.381	2.77 × 10^−7^	6.5578	1.84 × 10^−6^	1.40	0.49
Valine	−4.5325	6.88 × 10^−5^	4.1626	1.91 × 10^−4^	1.39	0.48
Homocysteine	−5.0377	1.53 × 10^−5^	4.8139	5.76 × 10^−5^	1.36	0.45
5-Methoxytryptamine	−3.1881	3.07 × 10^−3^	2.5129	6.22 × 10^−3^	1.34	0.42
Dopamine	−4.0565	2.75 × 10^−4^	3.5602	7.12 × 10^−4^	1.34	0.42
Arginine	−3.8544	4.90 × 10^−4^	3.3094	1.23 × 10^−3^	1.32	0.40
N-Acetylaspartic acid	−3.4377	1.57 × 10^−3^	2.805	3.36 × 10^−3^	1.30	0.38
Threonine	−3.555	1.14 × 10^−3^	2.945	2.66 × 10^−3^	1.27	0.35
2-Aminobutyric acid	−4.3809	1.07 × 10^−4^	3.9693	2.87 × 10^−4^	1.27	0.35
Glutarate	−2.7551	9.36 × 10^−3^	2.0288	1.71 × 10^−2^	1.26	0.33
Galacturonate	−3.5312	1.21 × 10^−3^	2.9165	2.75 × 10^−3^	1.25	0.32
Glucuronate	−3.4451	1.54 × 10^−3^	2.8137	3.36 × 10^−3^	1.24	0.31
2-Hydroxyglutarate	−3.0423	4.50 × 10^−3^	2.3465	8.89 × 10^−3^	1.22	0.28
Ribitol	2.4741	1.85 × 10^−2^	1.7327	3.30 × 10^−2^	0.87	−0.20
Tryptamine	3.3713	1.88 × 10^−3^	2.7264	3.91 × 10^−3^	0.76	−0.40
Fumarate	5.6446	2.50 × 10^−6^	5.6029	1.10 × 10^−5^	0.74	−0.44
Mannitol	7.0852	3.49 × 10^−8^	7.4577	3.29 × 10^−7^	0.72	−0.48
Sorbitol	7.0825	3.51 × 10^−8^	7.4543	3.29 × 10^−7^	0.71	−0.49
2-Ketoglutarate	5.549	3.32 × 10^−6^	5.4786	1.38 × 10^−5^	0.71	−0.49
Ribose	5.8504	1.35 × 10^−6^	5.8707	6.73 × 10^−6^	0.70	−0.51
Ribose 5-phosphate	6.0891	6.60 × 10^−7^	6.1804	3.54 × 10^−6^	0.70	−0.52
Xylulose	7.4246	1.30 × 10^−8^	7.8847	1.63 × 10^−7^	0.63	−0.66
Fructose 1-phosphate	5.519	3.63 × 10^−6^	5.4395	1.43 × 10^−5^	0.60	−0.73
Malate	3.7919	5.85 × 10^−4^	3.2325	1.42 × 10^−3^	0.59	−0.76
Mannose 6-phosphate	8.2876	1.13 × 10^−9^	8.9458	1.87 × 10^−8^	0.55	−0.85
Glucose 6-phosphate	8.2537	1.24 × 10^−9^	8.9049	1.87 × 10^−8^	0.55	−0.86
Ribulose 5-phosphate	4.8626	2.59 × 10^−5^	4.5872	8.44 × 10^−5^	0.47	−1.08
Dihydroxyacetone phosphate	21.487	2.31 × 10^−21^	20.636	8.68 × 10^−20^	0.32	−1.63

FDR: false discovery rate; log_2_(FC): log_2_(fold change).

**Table 2:** List of significant metabolites identified in HU210-treated cells compared with the control group (n = 58).

Metabolites	t-Stat	*p*-Value	−log_10_(p)	FDR	Fold Change	log_2_(FC)
Guanosine	−11.296	4.70 × 10^−13^	12.328	5.88 × 10^−12^	8.15	3.03
Glycerol 2-phosphate	−17.309	1.97 × 10^−18^	17.705	1.48 × 10^−16^	4.76	2.25
Glycerol 3-phosphate	−16.702	5.87 × 10^−18^	17.231	2.20 × 10^−16^	4.68	2.23
4-Aminobutyrate	−13.256	5.44 × 10^−15^	14.264	1.36 × 10^−13^	2.40	1.26
2-Ketoglutarate	−12.925	1.12 × 10^−14^	13.95	2.10 × 10^−13^	2.05	1.04
2-Hydroxyglutarate	−11.715	1.75 × 10^−13^	12.758	2.62 × 10^−12^	1.79	0.84
Ribose	−5.845	1.37 × 10^−6^	5.8635	6.04 × 10^−6^	1.77	0.83
Maleate	−9.0045	1.59 × 10^−10^	9.7978	1.19 × 10^−9^	1.67	0.74
N-Acetylserine	−9.9495	1.33 × 10^−11^	10.877	1.24 × 10^−10^	1.67	0.74
Ribitol	−10.428	3.95 × 10^−12^	11.404	4.23 × 10^−11^	1.61	0.68
Fumarate	−9.6501	2.88 × 10^−11^	10.54	2.40 × 10^−10^	1.59	0.67
Fructose 1-phosphate	−5.7665	1.73 × 10^−6^	5.7615	7.22 × 10^−6^	1.51	0.60
Creatinine	−3.8951	4.37 × 10^−4^	3.3595	1.06 × 10^−3^	1.51	0.59
N-Acetylaspartic acid	−6.4609	2.18 × 10^−7^	6.6606	1.17 × 10^−6^	1.48	0.56
Inositol phosphate	−4.8117	3.01 × 10^−5^	4.5215	9.41 × 10^−5^	1.47	0.56
Xylulose	−8.0247	2.36 × 10^−9^	8.6266	1.55 × 10^−8^	1.41	0.49
Galacturonate	−8.0081	2.48 × 10^−9^	8.6062	1.55 × 10^−8^	1.40	0.49
Glucuronate	−7.9317	3.07 × 10^−9^	8.5128	1.77 × 10^−8^	1.40	0.49
Homocysteine	−6.0063	8.45 × 10^−7^	6.073	3.96 × 10^−6^	1.34	0.42
1,6-Anhydroglucose	−3.6354	9.08 × 10^−4^	3.0419	2.13 × 10^−3^	1.34	0.42
Ribose 5-phosphate	−4.9526	1.98 × 10^−5^	4.7036	7.07 × 10^−5^	1.30	0.38
Pantothenate	−5.0598	1.44 × 10^−5^	4.8425	5.39 × 10^−5^	1.30	0.38
Malate	−3.4708	1.43 × 10^−3^	2.8443	2.90 × 10^−3^	1.30	0.38
Batyl alcohol	−5.6944	2.15 × 10^−6^	5.6678	8.48 × 10^−6^	1.27	0.34
Glucarate	−4.9022	2.30 × 10^−5^	4.6384	7.84 × 10^−5^	1.27	0.34
Methionine sulfone	−2.5502	1.54 × 10^−2^	1.8114	2.36 × 10^−2^	1.26	0.33
Gluconate	−4.6453	4.93 × 10^−5^	4.3072	1.42 × 10^−4^	1.23	0.30
3-Aminopropanoate	−3.4192	1.65 × 10^−3^	2.783	3.17 × 10^−3^	1.22	0.29
Arabinose	−2.4007	2.20 × 10^−2^	1.6579	3.09 × 10^−2^	1.22	0.28
Ribulose 5-phosphate	−2.555	1.53 × 10^−2^	1.8163	2.36 × 10^−2^	1.21	0.28
Asparagine	−2.5989	1.37 × 10^−2^	1.8623	2.29 × 10^−2^	1.20	0.26
Aconitate	−2.482	1.82 × 10^−2^	1.7408	2.69 × 10^−2^	1.20	0.26
2-Aminobutyrate	−3.4236	1.63 × 10^−3^	2.7882	3.17 × 10^−3^	1.20	0.26
Norepinephrine	−3.6108	9.72 × 10^−4^	3.0122	2.21 × 10^−3^	1.19	0.24
Phenylalanine	−2.677	1.14 × 10^−2^	1.945	1.93 × 10^−2^	1.17	0.23
Octopamine	−2.9386	5.89 × 10^−3^	2.2302	1.05 × 10^−2^	1.17	0.22
Valine	−2.5649	1.49 × 10^−2^	1.8266	2.36 × 10^−2^	1.14	0.19
Dihydroxyacetone phosphate	−2.4795	1.83 × 10^−2^	1.7382	2.69 × 10^−2^	1.13	0.18
Orotate	−2.262	3.02 × 10^−2^	1.52	4.04 × 10^−2^	1.13	0.18
Methionine	−2.2308	3.24 × 10^−2^	1.4895	4.26 × 10^−2^	1.12	0.17
Threonine	−2.5843	1.42 × 10^−2^	1.8469	2.32 × 10^−2^	1.12	0.17
Isoleucine	−2.1627	3.77 × 10^−2^	1.4238	4.87 × 10^−2^	1.11	0.15
2-Aminopimelate	2.9316	5.99 × 10^−3^	2.2224	1.05 × 10^−2^	0.84	−0.25
Tryptamine	2.4201	2.10 × 10^−2^	1.6776	3.03 × 10^−2^	0.83	−0.28
Dopamine	2.3486	2.48 × 10^−2^	1.6056	3.38 × 10^−2^	0.82	−0.28
Sorbitol	3.4901	1.36 × 10^−3^	2.8673	2.87 × 10^−3^	0.81	−0.30
Mannitol	3.4848	1.38 × 10^−3^	2.861	2.87 × 10^−3^	0.81	−0.30
Aspartate	4.8794	2.46 × 10^−5^	4.609	8.02 × 10^−5^	0.74	−0.43
Serine	2.9467	5.77 × 10^−3^	2.2392	1.05 × 10^−2^	0.74	−0.44
Citrate	3.1293	3.59 × 10^−3^	2.4454	6.72 × 10^−3^	0.66	−0.61
N-Acetylglutamine	4.6884	4.34 × 10^−5^	4.3627	1.30 × 10^−4^	0.62	−0.69
Nonanoate	3.5629	1.11 × 10^−3^	2.9545	2.45 × 10^−3^	0.62	−0.70
Fructose	4.2049	1.79 × 10^−4^	3.7464	4.98 × 10^−4^	0.61	−0.72
Mannose	4.035	2.93 × 10^−4^	3.5334	7.32 × 10^−4^	0.61	−0.72
Glucose	4.0406	2.88 × 10^−4^	3.5403	7.32 × 10^−4^	0.61	−0.72
Galactose	4.1182	2.30 × 10^−4^	3.6374	6.17 × 10^−4^	0.60	−0.73
Ornithine	2.3958	2.22 × 10^−2^	1.653	3.09 × 10^−2^	0.59	−0.77
Putrescine	6.2306	4.33 × 10^−7^	6.3636	2.16 × 10^−6^	0.54	−0.90

FDR: false discovery rate; log_2_(FC): log_2_(fold change).

**Table 3:** List of significant proteins identified in *Cannabis* extract-treated cells compared with the control group (n = 8).

Gene Name	t-Stat	*p*-Value	−log_10_(p)	FDR	Fold Change	log_2_(FC)
Hyou1	−6.1069	1.15 × 10^−4^	3.9407	3.90 × 10^−2^	1.02	0.03
Ddb1	6.4719	7.15 × 10^−5^	4.1459	3.24 × 10^−2^	0.94	−0.10
Atp1a1	8.5678	6.43 × 10^−6^	5.192	4.49 × 10^−3^	0.90	−0.15
Sqstm1	6.1281	1.11 × 10^−4^	3.9528	3.90 × 10^−2^	0.78	−0.36
Wdr6	8.5411	6.61 × 10^−6^	5.18	4.49 × 10^−3^	0.68	−0.57
Fau	6.8797	4.30 × 10^−5^	4.3666	2.34 × 10^−2^	0.67	−0.57
Hmgcs1	12.551	1.91 × 10^−7^	6.7184	5.20 × 10^−4^	0.65	−0.62
Rpl29	10.476	1.01 × 10^−6^	5.9846	1.41 × 10^−3^	0.63	−0.66

FDR: false discovery rate; log_2_(FC): log_2_(fold change).

**Table 4:** List of significant proteins identified in HU210-treated cells compared with the control group (n = 186).

Gene Name	t-Stat	*p*-Value	−log_10_(p)	FDR	Fold Change	log_2_(FC)
Camk2b	−3.9832	2.59 × 10^−3^	2.5871	4.51 × 10^−2^	1.90	0.92
App	−4.8908	6.32 × 10^−4^	3.1995	2.99 × 10^−2^	1.58	0.66
Serpini1	−4.207	1.81 × 10^−3^	2.7427	3.99 × 10^−2^	1.55	0.64
Aplp2	−4.9679	5.63 × 10^−4^	3.2492	2.99 × 10^−2^	1.52	0.60
Cst3	−7.7529	1.55 × 10^−5^	4.8102	7.80 × 10^−3^	1.49	0.58
Pgr	−4.4834	1.17 × 10^−3^	2.9307	3.46 × 10^−2^	1.46	0.55
Wfs1	−4.8485	6.73 × 10^−4^	3.1721	2.99 × 10^−2^	1.45	0.53
Htra1	−4.331	1.49 × 10^−3^	2.8276	3.63 × 10^−2^	1.43	0.51
Itga6	−4.251	1.69 × 10^−3^	2.7729	3.86 × 10^−2^	1.41	0.50
Pcsk1n	−5.2569	3.70 × 10^−4^	3.4321	2.29 × 10^−2^	1.41	0.50
Ddah2	−4.5548	1.05 × 10^−3^	2.9786	3.40 × 10^−2^	1.37	0.45
Stard5	−5.0675	4.87 × 10^−4^	3.3128	2.88 × 10^−2^	1.36	0.44
Scg5	−4.4272	1.28 × 10^−3^	2.8929	3.46 × 10^−2^	1.34	0.42
Gnao1	−4.2279	1.75 × 10^−3^	2.7571	3.92 × 10^−2^	1.33	0.42
Tppp3	−4.3818	1.37 × 10^−3^	2.8622	3.55 × 10^−2^	1.31	0.39
Cga	−5.2547	3.71 × 10^−4^	3.4307	2.29 × 10^−2^	1.30	0.38
Eif4ebp1	−7.5024	2.06 × 10^−5^	4.6867	8.64 × 10^−3^	1.29	0.36
B2m	−7.1871	2.97 × 10^−5^	4.5271	8.64 × 10^−3^	1.28	0.35
Lactb2	−4.2467	1.70 × 10^−3^	2.77	3.86 × 10^−2^	1.27	0.34
Gstm5	−4.9042	6.19 × 10^−4^	3.2082	2.99 × 10^−2^	1.26	0.33
Grhpr	−3.9115	2.91 × 10^−3^	2.5367	4.82 × 10^−2^	1.26	0.33
Dguok	−6.3264	8.61 × 10^−5^	4.065	1.33 × 10^−2^	1.24	0.31
Ppt1	−4.716	8.21 × 10^−4^	3.0854	3.18 × 10^−2^	1.23	0.30
Scg2	−7.2587	2.73 × 10^−5^	4.5638	8.64 × 10^−3^	1.23	0.29
Cox6c	−3.8817	3.05 × 10^−3^	2.5156	4.96 × 10^−2^	1.22	0.29
Atp6ap2	−5.4356	2.87 × 10^−4^	3.5427	2.24 × 10^−2^	1.22	0.28
Selenof	−4.8452	6.76 × 10^−4^	3.1699	2.99 × 10^−2^	1.21	0.28
Pcolce	−4.5205	1.11 × 10^−3^	2.9556	3.40 × 10^−2^	1.21	0.28
Hdac6	−4.334	1.48 × 10^−3^	2.8296	3.63 × 10^−2^	1.21	0.27
Pgm5	−4.1903	1.86 × 10^−3^	2.7312	4.03 × 10^−2^	1.21	0.27
Mt-co2	−5.9439	1.42 × 10^−4^	3.8465	1.66 × 10^−2^	1.20	0.26
Cox7a2	−7.2164	2.87 × 10^−5^	4.5421	8.64 × 10^−3^	1.20	0.26
Dhrs7b	−4.8387	6.83 × 10^−4^	3.1657	2.99 × 10^−2^	1.19	0.25
Cpq	−4.0739	2.24 × 10^−3^	2.6505	4.22 × 10^−2^	1.19	0.25
Fkbp3	−4.0491	2.33 × 10^−3^	2.6332	4.34 × 10^−2^	1.18	0.24
Adpgk	−4.2065	1.81 × 10^−3^	2.7424	3.99 × 10^−2^	1.18	0.24
Nipsnap1	−3.9448	2.75 × 10^−3^	2.5602	4.65 × 10^−2^	1.17	0.23
Mpdu1	−4.9432	5.84 × 10^−4^	3.2333	2.99 × 10^−2^	1.17	0.23
Txndc5	−4.3214	1.51 × 10^−3^	2.8211	3.63 × 10^−2^	1.17	0.23
Cntn1	−5.3842	3.08 × 10^−4^	3.5111	2.24 × 10^−2^	1.17	0.22
Gpt2	−4.0028	2.51 × 10^−3^	2.6009	4.48 × 10^−2^	1.17	0.22
Mthfd2	−4.5394	1.08 × 10^−3^	2.9683	3.40 × 10^−2^	1.16	0.21
Tlr13	−6.2563	9.43 × 10^−5^	4.0256	1.35 × 10^−2^	1.16	0.21
Etfdh	−4.3308	1.49 × 10^−3^	2.8275	3.63 × 10^−2^	1.15	0.20
Sfxn1	−6.0651	1.21 × 10^−4^	3.9166	1.53 × 10^−2^	1.15	0.20
Uqcrh	−4.3196	1.51 × 10^−3^	2.8198	3.63 × 10^−2^	1.15	0.20
Slc25a12	−4.5177	1.11 × 10^−3^	2.9538	3.40 × 10^−2^	1.15	0.20
Cox5b	−3.9808	2.60 × 10^−3^	2.5854	4.51 × 10^−2^	1.14	0.19
Psph	−5.5723	2.37 × 10^−4^	3.626	2.04 × 10^−2^	1.14	0.19
Pck2	−4.4148	1.30 × 10^−3^	2.8845	3.46 × 10^−2^	1.14	0.19
Slc25a20	−4.147	1.99 × 10^−3^	2.7013	4.04 × 10^−2^	1.14	0.19
Snx2	−4.6371	9.26 × 10^−4^	3.0333	3.26 × 10^−2^	1.14	0.19
Acads	−4.7176	8.19 × 10^−4^	3.0865	3.18 × 10^−2^	1.14	0.19
Cyb5r3	−4.748	7.83 × 10^−4^	3.1065	3.18 × 10^−2^	1.13	0.18
Prdx6	−4.5969	9.85 × 10^−4^	3.0066	3.33 × 10^−2^	1.13	0.18
Macroh2a1	−3.909	2.92 × 10^−3^	2.5349	4.82 × 10^−2^	1.13	0.17
Gdi1	−4.4091	1.32 × 10^−3^	2.8806	3.46 × 10^−2^	1.13	0.17
Smpdl3b	−4.1631	1.94 × 10^−3^	2.7124	4.04 × 10^−2^	1.12	0.17
Acot9	−4.3305	1.49 × 10^−3^	2.8273	3.63 × 10^−2^	1.12	0.17
Tmed4	−4.5248	1.10 × 10^−3^	2.9586	3.40 × 10^−2^	1.12	0.17
Anxa5	−4.1459	1.99 × 10^−3^	2.7005	4.04 × 10^−2^	1.12	0.17
Cstb	−3.949	2.73 × 10^−3^	2.5631	4.65 × 10^−2^	1.12	0.17
Cltb	−4.1643	1.94 × 10^−3^	2.7132	4.04 × 10^−2^	1.12	0.16
Cox5a	−5.771	1.80 × 10^−4^	3.7449	1.81 × 10^−2^	1.12	0.16
Cox4i1	−6.8492	4.46 × 10^−5^	4.3504	8.64 × 10^−3^	1.12	0.16
Aldh1l2	−4.4593	1.22 × 10^−3^	2.9146	3.46 × 10^−2^	1.11	0.16
Ctsd	−4.3677	1.40 × 10^−3^	2.8526	3.60 × 10^−2^	1.11	0.15
Mpst	−4.0957	2.16 × 10^−3^	2.6657	4.18 × 10^−2^	1.11	0.15
Hk1	−4.7768	7.49 × 10^−4^	3.1253	3.18 × 10^−2^	1.11	0.15
Coxfa4	−6.8716	4.34 × 10^−5^	4.3623	8.64 × 10^−3^	1.11	0.15
Sars1	−4.2954	1.57 × 10^−3^	2.8033	3.69 × 10^−2^	1.11	0.15
Prpsap2	−4.9083	6.15 × 10^−4^	3.2108	2.99 × 10^−2^	1.11	0.15
Pygb	−3.9481	2.74 × 10^−3^	2.5624	4.65 × 10^−2^	1.11	0.15
Hexb	−5.3522	3.23 × 10^−4^	3.4914	2.24 × 10^−2^	1.11	0.15
Pgrmc1	−4.5698	1.03 × 10^−3^	2.9886	3.40 × 10^−2^	1.11	0.15
Hyou1	−4.0925	2.17 × 10^−3^	2.6635	4.18 × 10^−2^	1.11	0.14
Idh3a	−4.5467	1.06 × 10^−3^	2.9732	3.40 × 10^−2^	1.10	0.14
Sec11a	−4.1817	1.88 × 10^−3^	2.7253	4.03 × 10^−2^	1.10	0.14
Bpnt1	−3.8734	3.09 × 10^−3^	2.5098	5.00 × 10^−2^	1.10	0.14
Got1	−5.3682	3.15 × 10^−4^	3.5012	2.24 × 10^−2^	1.10	0.13
Hsd17b4	−4.0122	2.47 × 10^−3^	2.6074	4.47 × 10^−2^	1.09	0.13
Ppa1	−4.5926	9.91 × 10^−4^	3.0038	3.33 × 10^−2^	1.09	0.13
Pgd	−4.7346	7.99 × 10^−4^	3.0977	3.18 × 10^−2^	1.09	0.13
Manf	−6.9154	4.12 × 10^−5^	4.3855	8.64 × 10^−3^	1.09	0.13
Prkcsh	−3.9444	2.76 × 10^−3^	2.5598	4.65 × 10^−2^	1.09	0.13
Mdh1	−4.3499	1.44 × 10^−3^	2.8405	3.63 × 10^−2^	1.09	0.13
Cyb5r1	−4.109	2.11 × 10^−3^	2.6749	4.12 × 10^−2^	1.09	0.13
Pdia3	−3.9598	2.69 × 10^−3^	2.5707	4.64 × 10^−2^	1.09	0.13
Gstp1	−5.3588	3.20 × 10^−4^	3.4954	2.24 × 10^−2^	1.09	0.13
Aldh18a1	−4.4198	1.29 × 10^−3^	2.8879	3.46 × 10^−2^	1.09	0.13
Atox1	−4.6129	9.61 × 10^−4^	3.0173	3.33 × 10^−2^	1.09	0.12
Plod3	−7.9465	1.25 × 10^−5^	4.9035	7.80 × 10^−3^	1.09	0.12
Prdx2	−4.0197	2.44 × 10^−3^	2.6127	4.44 × 10^−2^	1.09	0.12
Decr1	−4.3079	1.54 × 10^−3^	2.8119	3.67 × 10^−2^	1.08	0.11
Naxe	−4.6778	8.70 × 10^−4^	3.0602	3.22 × 10^−2^	1.08	0.11
Dnpep	−5.2379	3.80 × 10^−4^	3.4203	2.30 × 10^−2^	1.08	0.11
Rpn1	−4.7296	8.05 × 10^−4^	3.0944	3.18 × 10^−2^	1.08	0.11
Akr1a1	−4.9493	5.79 × 10^−4^	3.2373	2.99 × 10^−2^	1.08	0.11
Septin9	−4.0852	2.20 × 10^−3^	2.6584	4.19 × 10^−2^	1.07	0.10
Cnpy2	−5.0369	5.09 × 10^−4^	3.2933	2.96 × 10^−2^	1.07	0.10
Isoc1	−4.9468	5.81 × 10^−4^	3.2356	2.99 × 10^−2^	1.07	0.10
Hnrnpul2	−4.8571	6.64 × 10^−4^	3.1777	2.99 × 10^−2^	1.07	0.10
Nars1	−4.495	1.15 × 10^−3^	2.9386	3.46 × 10^−2^	1.06	0.09
Iars1	−4.7462	7.85 × 10^−4^	3.1053	3.18 × 10^−2^	1.06	0.09
Rbmxl1	−3.8886	3.02 × 10^−3^	2.5205	4.93 × 10^−2^	1.06	0.08
Dlst	−4.1104	2.11 × 10^−3^	2.6759	4.12 × 10^−2^	1.06	0.08
Ogdh	−4.0308	2.40 × 10^−3^	2.6204	4.42 × 10^−2^	1.06	0.08
Mrpl12	−4.4119	1.31 × 10^−3^	2.8826	3.46 × 10^−2^	1.06	0.08
Uqcrc2	−4.1803	1.89 × 10^−3^	2.7243	4.03 × 10^−2^	1.06	0.08
Ilf3	−4.6432	9.18 × 10^−4^	3.0374	3.26 × 10^−2^	1.05	0.07
Ina	−4.1141	2.10 × 10^−3^	2.6785	4.12 × 10^−2^	1.05	0.07
Phb2	−4.3923	1.35 × 10^−3^	2.8693	3.52 × 10^−2^	1.05	0.07
Pgk1	−4.6749	8.74 × 10^−4^	3.0584	3.22 × 10^−2^	1.05	0.07
Mdh2	−6.943	3.98 × 10^−5^	4.4	8.64 × 10^−3^	1.05	0.06
Lars1	−4.0351	2.38 × 10^−3^	2.6234	4.41 × 10^−2^	1.04	0.06
Dlat	−4.1849	1.87 × 10^−3^	2.7275	4.03 × 10^−2^	1.04	0.06
Etfb	−4.4413	1.25 × 10^−3^	2.9024	3.46 × 10^−2^	1.04	0.06
Ruvbl2	−4.5349	1.08 × 10^−3^	2.9653	3.40 × 10^−2^	1.04	0.05
Ddx1	−5.3906	3.05 × 10^−4^	3.515	2.24 × 10^−2^	1.03	0.05
Ahcy	−4.0047	2.50 × 10^−3^	2.6022	4.48 × 10^−2^	1.03	0.05
Mcm2	4.1601	1.95 × 10^−3^	2.7104	4.04 × 10^−2^	0.95	−0.07
Atp1a1	4.6519	9.05 × 10^−4^	3.0431	3.26 × 10^−2^	0.95	−0.07
Prrc2c	4.6937	8.50 × 10^−4^	3.0708	3.21 × 10^−2^	0.95	−0.07
Map1b	5.3448	3.26 × 10^−4^	3.4868	2.24 × 10^−2^	0.95	−0.07
Dnmt1	6.3912	7.92 × 10^−5^	4.1012	1.33 × 10^−2^	0.95	−0.07
Xpo5	3.8979	2.97 × 10^−3^	2.5271	4.88 × 10^−2^	0.95	−0.08
Tln1	4.5243	1.10 × 10^−3^	2.9582	3.40 × 10^−2^	0.95	−0.08
Eef2	6.128	1.12 × 10^−4^	3.9527	1.47 × 10^−2^	0.95	−0.08
Eif3a	3.9399	2.78 × 10^−3^	2.5567	4.66 × 10^−2^	0.94	−0.08
Pabpc1	4.449	1.24 × 10^−3^	2.9076	3.46 × 10^−2^	0.94	−0.09
Hdac2	5.7046	1.97 × 10^−4^	3.7054	1.92 × 10^−2^	0.94	−0.09
Brix1	4.138	2.02 × 10^−3^	2.6951	4.04 × 10^−2^	0.94	−0.09
Pgam5	4.7043	8.36 × 10^−4^	3.0777	3.20 × 10^−2^	0.94	−0.09
Nedd4	5.2781	3.59 × 10^−4^	3.4453	2.29 × 10^−2^	0.94	−0.09
Ddx3x	6.2222	9.86 × 10^−5^	4.0063	1.35 × 10^−2^	0.94	−0.09
Plxnb2	4.255	1.68 × 10^−3^	2.7756	3.86 × 10^−2^	0.94	−0.09
Rpl9	4.8128	7.10 × 10^−4^	3.1488	3.07 × 10^−2^	0.94	−0.10
Prpf19	3.9201	2.87 × 10^−3^	2.5427	4.79 × 10^−2^	0.93	−0.10
Eif3h	5.0174	5.24 × 10^−4^	3.2809	2.99 × 10^−2^	0.92	−0.11
Ctnnd1	4.4594	1.22 × 10^−3^	2.9146	3.46 × 10^−2^	0.92	−0.12
Grk2	4.3194	1.51 × 10^−3^	2.8197	3.63 × 10^−2^	0.92	−0.12
Atxn10	5.8575	1.60 × 10^−4^	3.7959	1.76 × 10^−2^	0.92	−0.12
Rpl30	5.6012	2.27 × 10^−4^	3.6434	2.02 × 10^−2^	0.92	−0.13
Ddb1	6.8297	4.57 × 10^−5^	4.34	8.64 × 10^−3^	0.92	−0.13
Zyx	3.9952	2.54 × 10^−3^	2.5955	4.51 × 10^−2^	0.91	−0.13
Tfip11	4.0523	2.32 × 10^−3^	2.6354	4.34 × 10^−2^	0.91	−0.14
Rrm1	4.9476	5.81 × 10^−4^	3.2361	2.99 × 10^−2^	0.91	−0.14
Psme3	4.1781	1.89 × 10^−3^	2.7227	4.03 × 10^−2^	0.90	−0.14
G3bp2	4.6005	9.79 × 10^−4^	3.0091	3.33 × 10^−2^	0.90	−0.15
Nme1	4.4905	1.16 × 10^−3^	2.9355	3.46 × 10^−2^	0.90	−0.15
Uhrf1	4.4801	1.18 × 10^−3^	2.9285	3.46 × 10^−2^	0.90	−0.15
Bpnt2	7.9736	1.21 × 10^−5^	4.9165	7.80 × 10^−3^	0.89	−0.16
Ilkap	4.1343	2.03 × 10^−3^	2.6925	4.04 × 10^−2^	0.89	−0.17
Rps7	4.157	1.96 × 10^−3^	2.7082	4.04 × 10^−2^	0.89	−0.17
Caprin1	6.3107	8.79 × 10^−5^	4.0562	1.33 × 10^−2^	0.88	−0.18
Tbl3	4.6429	9.18 × 10^−4^	3.0372	3.26 × 10^−2^	0.88	−0.18
Abcf2	5.9729	1.37 × 10^−4^	3.8634	1.66 × 10^−2^	0.88	−0.18
Tfrc	4.2474	1.70 × 10^−3^	2.7704	3.86 × 10^−2^	0.88	−0.19
Bag6	5.2896	3.53 × 10^−4^	3.4525	2.29 × 10^−2^	0.88	−0.19
Csde1	4.8566	6.65 × 10^−4^	3.1773	2.99 × 10^−2^	0.87	−0.20
Cab39	7.0053	3.69 × 10^−5^	4.4328	8.64 × 10^−3^	0.87	−0.20
Larp4	5.2873	3.54 × 10^−4^	3.4511	2.29 × 10^−2^	0.87	−0.21
Txndc9	4.0834	2.20 × 10^−3^	2.6571	4.19 × 10^−2^	0.86	−0.21
Glg1	4.7523	7.78 × 10^−4^	3.1093	3.18 × 10^−2^	0.86	−0.21
Supt6h	3.986	2.58 × 10^−3^	2.5891	4.51 × 10^−2^	0.86	−0.22
Tor1aip2	4.1364	2.02 × 10^−3^	2.6939	4.04 × 10^−2^	0.84	−0.24
Btf3	5.844	1.63 × 10^−4^	3.788	1.76 × 10^−2^	0.84	−0.25
Gtf2f2	5.4047	2.99 × 10^−4^	3.5237	2.24 × 10^−2^	0.84	−0.26
Cpd	4.4378	1.26 × 10^−3^	2.9	3.46 × 10^−2^	0.83	−0.26
Tmx2	7.9024	1.31 × 10^−5^	4.8824	7.80 × 10^−3^	0.83	−0.27
Vim	3.9849	2.58 × 10^−3^	2.5883	4.51 × 10^−2^	0.82	−0.29
Ubtf	4.9654	5.66 × 10^−4^	3.2476	2.99 × 10^−2^	0.81	−0.30
Tjp1	4.2313	1.74 × 10^−3^	2.7594	3.92 × 10^−2^	0.78	−0.36
Gclm	4.8837	6.38 × 10^−4^	3.1949	2.99 × 10^−2^	0.77	−0.37
Hmgcs1	10.012	1.57 × 10^−6^	5.8037	4.75 × 10^−3^	0.76	−0.40
Cfap36	4.4548	1.23 × 10^−3^	2.9115	3.46 × 10^−2^	0.76	−0.41
Sqstm1	5.4376	2.86 × 10^−4^	3.5439	2.24 × 10^−2^	0.69	−0.53
Wdr6	8.6746	5.75 × 10^−6^	5.24	7.80 × 10^−3^	0.64	−0.64
Thumpd3	5.8066	1.71 × 10^−4^	3.766	1.79 × 10^−2^	0.63	−0.66
Pak3	4.1377	2.02 × 10^−3^	2.6948	4.04 × 10^−2^	0.61	−0.72
Irs4	4.4296	1.28 × 10^−3^	2.8945	3.46 × 10^−2^	0.60	−0.74
Dock7	4.8453	6.76 × 10^−4^	3.17	2.99 × 10^−2^	0.56	−0.84
Hbb-b1	5.6594	2.10 × 10^−4^	3.6784	1.92 × 10^−2^	0.56	−0.84
Alb	4.2979	1.57 × 10^−3^	2.8051	3.69 × 10^−2^	0.48	−1.07
Slc39a8	4.0197	2.44 × 10^−3^	2.6127	4.44 × 10^−2^	0.48	−1.07
Hba	6.5147	6.77 × 10^−5^	4.1695	1.20 × 10^−2^	0.40	−1.33

FDR: false discovery rate; log_2_(FC): log_2_(fold change).

## Data Availability

Data are contained within the article or the Supplementary Materials.

## Supplementary Materials

The following supporting information can be downloaded at https://www.mdpi.com/article/10.3390/metabo16090645/s1, Supplementary Table S1. List of metabolites; Supplementary Table S2. List of differentially ex-pressed proteins; Supplementary Table S3. Significant pathways; Supplementary Table S4. CBD, THC, and THCA; Supplementary Table S5. LDH_results; Supplementary Table S6. Metabolites data set; Supplementary Table S7. Proteins data set; Supplementary Figure S1. Cannabinoids de-tected in CS; Supplementary Figure S2. Scheme of the proteomic analysis.

## Abbreviations

The following abbreviations are used in this manuscript:

ECS	Endocannabinoid system
CB	cannabinoid
CB1R	cannabinoid receptor type 1
CB2R	cannabinoid receptor type 2
GPCR	class A G protein-coupled receptor
CNS	central nervous system
AEA	*N*-arachidonoylethanolamine, anandamide
2-AG	2-arachidonoylglycerol
THC	Δ9-tetrahydrocannabinol
CBD	cannabidiol
TRPV1	transient receptor potential vanilloid 1
5-HT 1A	serotonin 1A receptor
HU	HU210
CS	*Cannabis sativa* extract
GPR55	G protein-coupled receptor 55
PCA	principal component analysis
Pck2	phosphoenolpyruvate carboxykinase
Idh3a	isocitrate dehydrogenase subunit α
Ogdh	2-oxoglutarate dehydrogenase complex component E1
Sdha	succinate dehydrogenase flavoprotein subunit a
Mdh1	malate dehydrogenase 1
Pgls	6-phosphogluconolactonase
Pgd	6-phosphogluconate dehydrogenase
Pgm1	phosphoglucomutase-1
PPP	pentose phosphate pathway
ROS	reactive oxygen species
ACO2	aconitase
THCV	tetrahydrocannabivarin
CBL	cannabicyclol
CBC	cannabicromene
THCA	tetrahydrocannabinolic acid
CBLA	cannabicyclolic acid
MAS	malate–aspartate shuttle
SREBP-1.	sterol regulatory element-binding protein 1
