# Supplementary material for: Integrated Metabolomic and Proteomic Analyses Reveal Differential Regulation of the Citrate Cycle and Pentose Phosphate Pathway by Cannabis sativa Extract and the Synthetic Cannabinoid HU210 in GT1-7 Neuronal Cells: Sequential Extraction of Metabolites and Proteins from a Single Cell Pellet
Source: Metabolites. 2026 Sep 3;16(9):645. doi: 10.3390/metabo16090645 (PMC13609593; doi:10.3390/metabo16090645)
Supplement: Supplementary file 1 [file metabolites-16-00645-s001.zip › Supplementary_Table_S1_List_of_metaboltites.pdf]

Supplementary Table S1

List of metabolites identified in this study (n = 75).

1,6-Anhydroglucose	Cysteine	Inositol phosphate	Putrescine
2-Aminobutyrate	Dihydroxyacetone phosphate	Isoleucine	Ribitol
2-Aminopimelate	Dopamine	Maleate	Ribose
2-Hydroxyglutarate	Fructose	Malate	Ribose 5-phosphate
2-Ketoglutarate	Fructose 1-phosphate	Mannitol	Ribulose 5-phosphate
3-Aminopropanoate	Fumarate	Mannose	Serine
4-Aminobutyrate	Galactose	Mannose 6-phosphate	Sorbitol
5-Aminovalerate	Galacturonate	meso-Erythritol	Tartarate
5-Methoxytryptamine	Glucarate	Methionine	Threitol
Aconitate	Gluconate	Methionine sulfone	Threonine
Arabinose	Glucose	N-Acetylaspartate	Triethanolamine
Arabitol	Glucose 6-phosphate	N-Acetylglutamine	Tryptamine
Arginine	Glucuronate	N-Acetylserine	Valine
Asparagine	Glutamate	Nonanoate	Xylose
Aspartate	Glutarate	Norepinephrine	Xylulose
Azelate	Glycerol 2-phosphate	Octopamine	
Batyl alcohol	Glycerol 3-phosphate	Ornithine	
Cadaverine	Guanosine	Orotate	
Citrate	Homocysteine	Pantothenate	
Creatinine	Hypotaurine	Phenylalanine	
