# Supplementary material for: Integrated Metabolomic and Proteomic Analyses Reveal Differential Regulation of the Citrate Cycle and Pentose Phosphate Pathway by Cannabis sativa Extract and the Synthetic Cannabinoid HU210 in GT1-7 Neuronal Cells: Sequential Extraction of Metabolites and Proteins from a Single Cell Pellet
Source: Metabolites. 2026 Sep 3;16(9):645. doi: 10.3390/metabo16090645 (PMC13609593; doi:10.3390/metabo16090645)
Supplement: Supplementary file 1 [file metabolites-16-00645-s001.zip › Supplementary_Table_S2_List of differentially expressed proteins.pdf]

Supplementary Table S2

List of differentially expressed proteins identified across the three groups (n = 267).

Abcf2	Btf3	Cyc1	Gnao1	Iars1	Nop2	Ppa1	Sdcbp	Tln1
Acads	Cab39	Cyp51a1	Gng4	Idh3a	Nucb1	Ppt1	Sdha	Tlr13
Acat2	Caprin1	Dbn1	Got1	Impdh1	Nup133	Prdx2	Sdk1	Tmed4
Acot9	Ccdc124	Dclk1	Gpam	Impdh2	Nup210	Prdx6	Sec11a	Tmsb10
Acox1	Cd81	Ddah2	Gpd2	Ina	Ogdh	Prkcsh	Sec22b	Tmx1
Acsl4	Cdh2	Ddb1	Grb14	Irs4	Osbp	Prpf19	Sec63	Tmx2
Adpgk	Celf6	Ddx3x	Grhpr	Itga6	Ostc	Prpf8	Septin5	Tppp3
Adss2	Cfap36	Ddx47	Grk2	Kras	Oxct1	Prpsap2	Septin9	Ttll12
Ahsg	Cga	Dnajc3	Gstp1	Larp4	Pabpc1	Psat1	Serpini1	Tuba3a
Aimp2	Cltb	Dnmt1	H1-0	Lars1	Pak3	Psph	Sfxn1	Tuba3b
Akr1a1	Cnpy2	Dnpep	H1-1	Lrrc41	Palld	Pvalb	Sh3gl2	Tubb2a
Alb	Cntn1	Dock7	Hba	Macroh2a1	Pck2	Rab3a	Shmt2	Txndc5
Aldh18a1	Cox4i1	Eef2	Hbb-b1	Manf	Pcna	Rbmxl1	Slc25a12	Txndc9
Aldh7a1	Cox5a	Eif3d	Hdac2	Map1b	Pcolce	Rpl14	Slc38a1	Ubl4a
Alg2	Cox6c	Eif4a1	Hdgfl3	Mars1	Pcsk1n	Rpl24	Slc39a8	Ubtf
Anxa5	Cox7a2	Eif4ebp1	Hexa	Mcm2	Pdia3	Rpl29	Slc3a2	Unc45a
Ap1g1	Coxfa4	Ergic1	Hexb	Mdh1	Pgam5	Rpl32	Snap25	Uqcrc1
App	Cpd	Etfb	Hibch	Micos10	Pgd	Rpl36a	Snrpb	Uqcrc2
Atox1	Cpq	Etfdh	Hk1	Morf4l1	Pgk1	Rpl6	Snx2	Uqcrq
Atp1a1	Csde1	Ethe1	Hkdc1	Mpdu1	Pgls	Rpn1	Sqstm1	Vapa
Atp2b1	Csnk2a1	Fads2	Hmgcs1	Mpst	Pgm1	Rps28	Srsf10	Vapb
Atp6ap2	Cst3	Faf1	Hnrnpul2	Mrfap1	Pgm5	Rps6	Srsf6	Vim
B2m	Cstb	Fau	Hp1bp3	Mt-co2	Pgp	Rps7	Ssb	Vps28
Bag6	Ctnnd1	Fdps	Hsd17b11	Mthfd2	Pgr	Rsl1d1	Stard5	Wdr43
Baiap2	Ctsb	Fkbp3	Hsd17b4	Nars1	Phb2	Ruvbl2	Stt3a	Wdr6
Bckdha	Ctsd	Foxl2	Hspa13	Nedd4	Plod3	Sars1	Syn2	Wfs1
Blmh	Ctsl	G3bp2	Hspa8	Neu1	Pogz	Scd2	Tfrc	Zdhhc13
Bpnt1	Cuedc2	Gclm	Hsph1	Nipsnap1	Pole	Scg2	Tgoln1	
Bpnt2	Cul4b	Gdi1	Htra1	Nme1	Polr2a	Scg5	Thumpd3	
Brix1	Cyb5r3	Glg1	Hyou1	Nomo1	Polr2g	Scpep1	Tkfc	
