# Supplementary material for: Integrated Metabolomic and Proteomic Analyses Reveal Differential Regulation of the Citrate Cycle and Pentose Phosphate Pathway by Cannabis sativa Extract and the Synthetic Cannabinoid HU210 in GT1-7 Neuronal Cells: Sequential Extraction of Metabolites and Proteins from a Single Cell Pellet
Source: Metabolites. 2026 Sep 3;16(9):645. doi: 10.3390/metabo16090645 (PMC13609593; doi:10.3390/metabo16090645)
Supplement: Supplementary file 1 [file metabolites-16-00645-s001.zip › Supplementary_Table_S3_Significant_pathways.pdf]

Supplementary Table S3

Total component number, metabolite and protein hits number, Raw *p*, $-\log_{10}(p)$, Holm adjust, FDR, and Impact values of significant pathways based on pathway score.

	Total	Hits.met	Hits.pro	Raw *p*	$-\log_{10}(p)$	Holm adjust	FDR	Impact
Citrate cycle (TCA cycle)	42	4	5	0.00028145	3.5506	0.016887	0.0039966	0.97561
Pyruvate metabolism	48	2	6	0.0058919	2.2297	0.2887	0.024746	0.68085
Pentose phosphate pathway	49	4	3	0.015816	1.8009	0.69589	0.049819	0.66667
Galactose metabolism	51	5	3	0.0031696	2.499	0.16165	0.015361	0.66
Butanoate metabolism	29	3	4	0.00068349	3.1653	0.039642	0.0071766	0.64286
Glycerolipid metabolism	34	2	4	0.011416	1.9425	0.53654	0.042306	0.48485

Hits of metabolites, Hits.met; Hits protein, Hits.pro; false-discovery rate, FDR
